# Manipulating local coordination of copper single atom catalyst enables efficient CO_2_-to-CH_4_ conversion

**DOI:** 10.1038/s41467-023-39048-6

**Published:** 2023-06-08

**Authors:** Yizhou Dai, Huan Li, Chuanhao Wang, Weiqing Xue, Menglu Zhang, Donghao Zhao, Jing Xue, Jiawei Li, Laihao Luo, Chunxiao Liu, Xu Li, Peixin Cui, Qiu Jiang, Tingting Zheng, Songqi Gu, Yao Zhang, Jianping Xiao, Chuan Xia, Jie Zeng

**Affiliations:** 1 Hefei National Research Center for Physical Sciences at the Microscale, University of Science and Technology of China, 230026 Hefei, Anhui P. R. China; 2 School of Materials and Energy, University of Electronic Science and Technology of China, 611731 Chengdu, P. R. China; 3 State Key Laboratory of Catalysis, Dalian Institute of Chemical Physics, Dalian National Laboratory for Clean Energy, Chinese Academy of Sciences, 116023 Dalian, P. R. China; 4 University of Chinese Academy of Sciences, 100049 Beijing, P. R. China; 5 Key Laboratory of Soil Environment and Pollution Remediation, Institute of Soil Science, Chinese Academy of Sciences, 210008 Nanjing, P. R. China; 6 Shanghai Advanced Research Institute, Chinese Academy of Sciences, 201210 Shanghai, P. R. China; 7 Yangtze Delta Region Institute (Huzhou), University of Electronic Science and Technology of China, 313001 Huzhou, Zhejiang China; 8 School of Chemistry & Chemical Engineering, Anhui University of Technology, 243002 Ma’anshan, Anhui P. R. China

**Keywords:** Electrocatalysis, Materials for energy and catalysis, Electrocatalysis

## Abstract

Electrochemical CO_2_ conversion to methane, powered by intermittent renewable electricity, provides an entrancing opportunity to both store renewable electric energy and utilize emitted CO_2_. Copper-based single atom catalysts are promising candidates to restrain C-C coupling, suggesting feasibility in further protonation of CO* to CHO* for methane production. In theoretical studies herein, we find that introducing boron atoms into the first coordination layer of Cu-N_4_ motif facilitates the binding of CO* and CHO* intermediates, which favors the generation of methane. Accordingly, we employ a co-doping strategy to fabricate B-doped Cu-N_*x*_ atomic configuration (Cu-N_*x*_B_*y*_), where Cu-N_2_B_2_ is resolved to be the dominant site. Compared with Cu-N_4_ motifs, as-synthesized B-doped Cu-N_*x*_ structure exhibits a superior performance towards methane production, showing a peak methane Faradaic efficiency of 73% at −1.46 V *vs*. RHE and a maximum methane partial current density of −462 mA cm^−2^ at −1.94 V *vs*. RHE. Extensional calculations utilizing two-dimensional reaction phase diagram analysis together with barrier calculation help to gain more insights into the reaction mechanism of Cu-N_2_B_2_ coordination structure.

## Abstract (web summary)

Developing efficient electrocatalysts for selective CO2 conversion is of high interest. Here the authors investigate B doped Cu single atom catalysts with Cu-N2B2 coordination for enhanced CO2 to CH4 conversion.

## Introduction

The immoderate burning of fossil fuels along with the wanton emission of carbon dioxide (CO_2_) into atmosphere has aroused the global warming effect^1–4^. Electrocatalytic CO_2_ conversion (CO_2_RR), powered by intermittent renewable electricity, provides an unprecedented possibility to address this global challenge^5–8^. Previous studies on CO_2_RR have reported the generation of various C_1_-C_3_ products including hydrocarbons and oxygenates^9–17^. Methane (CH_4_) possesses the largest heating value among hydrocarbons and is an important raw material for the manufacture of many other chemical products including aromatic hydrocarbon. Also, CH_4_ as the main component of natural gas, possesses a good compatibility with the existing infrastructure for storage, distribution, and consumption^18–20^. Then, electrocatalytic conversion of CO_2_ into CH_4_ offers an entrancing opportunity to both storing renewable electric energy and utilizing CO_2_ emissions. In general, starting with the CO* intermediates of CO_2_RR, protonation of CO* to CHO* leads to CH_4_, whereas the competitive CO* dimerization generates C_2_ products^21–24^. On the other hand, the non-optimized binding of CO* intermediates will result in release of gaseous CO, further suppressing the CH_4_ selectivty^25^. To perform CO_2_-to-CH_4_ economically at scale, a catalyst capable of mediating the efficient formation of CH_4_ with high selectivity and productivity is a prerequisite. Copper (Cu) has been extensively noted for their high catalytic activity towards hydrocarbons for CO_2_RR^26–29^. However, selective production of CH_4_ using Cu catalysts can be difficult owing to the sluggish eight electron transfer steps of CO_2_-to-CH_4_ and the inevitable C-C coupling process on bulk Cu^30,31^.

Single-atom catalysts (SACs) with adjustable and isolated active sites have been applied to electrochemical catalysis^32–34^, showing potential in expelling C-C coupling in CO_2_RR. Particularly, the unique 3*d* transition-metal-four nitrogen (M-N_4_) configuration consisting of an isolated single metal atom coordinated by four N atoms in carbon matrix has been demonstrated to be favorable for CO_2_ electroreduction^13,25,35^. However, the performance of Cu-N_4_ materials in methane production is still unsatisfactory, specifically, manifesting selectivity to CO at a less cathodic potential (<−1.0 V versus reversible hydrogen electrode, vs. RHE)^36–38^ and sluggish kinetics to CH_4_ at a more cathodic potential^39^. Previous theoretical calculations revealed that relatively weak adsorption of CO* over Cu-N_4_ results in feasible CO* desorption instead of further protonation to CH_4_^40^. As such, modulation of the electronic structure of Cu-N_4_ sites, by substituting relatively weak electronegativity functionalities, e.g., boron (B), is promising to facilitate the adsorption of key intermediates and thus steer the CO_2_RR selectivity towards CH_4_.

Here, we firstly conducted theoretical simulations to predict the adsorption of intermediates and thermodynamic trend over a series of sites with different concentration of boron in Cu-N_*x*_B_*y*_. It showed that, by introducing boron atoms into the coordination structure of Cu-N_4_, the binding of CO* and CHO* intermediates are promoted significantly, indicating the facile generation of CH_4_. Inspired by the theoretical predictions, we then managed to manipulate the nearest neighbor structure of isolated Cu sites with boron dopant (BNC-Cu). Comprehensive analysis of XAS data revealed that atomically dispersed Cu atoms in BNC-Cu possessed the B-doped Cu-N_*x*_ structure (Cu-N_*x*_B_*y*_) mainly in the form of Cu-N_2_B_2_. Electrochemical measurements displayed a huge boost in CH_4_ production, with BNC-Cu showing a high CH_4_ Faradaic efficiency (FE) of 73% and partial CH_4_ current density (*j*_CH4_) of −292 mA cm^− 2^ at −1.46 V vs. RHE. The divergence in CO_2_RR performance between boron-doped and undoped Cu sites validated the theoretical predictions, demonstrating that doping B into Cu-N_4_ structure serves as an effective way to enhance deep reduction activity in CO_2_RR. We further studied several Cu-N_2_B_2_-containing sites with the application of two-dimensional (*quasi*) activity and selectivity map along with reaction phase diagram, and the microkinetic simulation of selectivity also matched well with experimental data.

## Results and discussion

### Thermodynamic trend prediction of CO_2_-to-CH_4_ on Cu-N_*x*_B_*y*_

We first built a series of Cu-N_*x*_B_*y*_ structures with different boron concentrations (Supplementary Fig. 1) and performed density functional theory (DFT) calculations to obtain the adsorption energies of all intermediates in CO_2_-to-CH_4_ (Supplementary Fig. 2). Based on the scaling relations shown in Supplementary Fig. 3, the adsorption free energy of intermediate CHO*, *G*_ad_(CHO*), was chosen as the primary descriptor to evaluate the (thermodynamic) activity trend of CO_2_ RR to CH_4_. The trend was established considering optimal paths with globally minimal (limiting) energies.

**Fig. 1:**
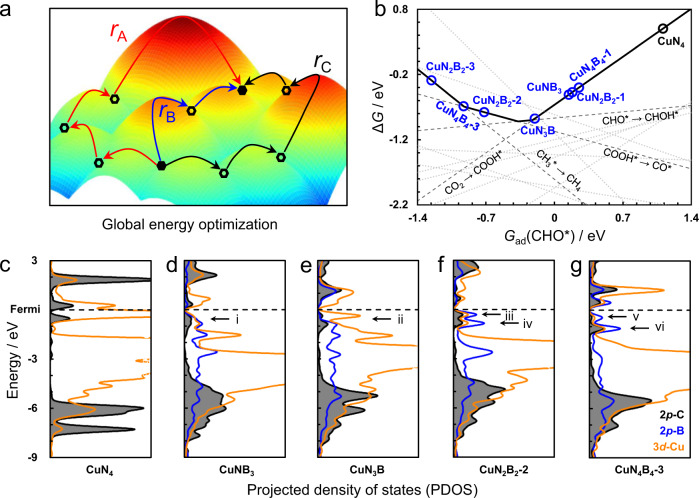
Theoretical prediction of activity on Cu-N_*x*_B_*y*_. **a** A scheme of global energy optimization to determine the optimal path. **b** Thermodynamic trend for CO_2_RR to CH_4_ at −1.2 V vs. RHE, as a function with *G*_ad_(CHO*). **c**–**f** Projected density of states (PDOS) for adsorbed CHO* over Cu-N_4_ (**c**), Cu-N_3_B (**d**), Cu-N_2_B_2_ (**e**), Cu-NB_3_ (**f**), and Cu-N_4_B_4_ (**g**), where the electronic states of 3*d*-Cu, 2*p*-C (of CHO*), and 2*p*-B are shown in orange, gray, and blue, respectively.

As schematically shown in Fig. 1a, a reaction path consists of many elementary steps. For given paths (black and red), they are not limited by the steps at the beginning of reaction. Instead, *r*_A_ and *r*_C_ are the limiting factors. All competing reaction paths were enumerated by the CatRPD code^41^, prior to energetic comparison, as explicitly described in our previous work^41,42^. Briefly, for the internal comparison of a path, the most difficult steps were first determined, such as the *r*_A_, *r*_B_, and *r*_C_ from these three different paths in Fig. 1a. Then, the external comparison between all paths can derive the optimal step and energy, defined as ∆*G*_RPD_-limiting step and energy. In this case, blue path consisting of *r*_B_ with the global minimum Δ*G* is considered as the actual path while *r*_B_ is ∆*G*_RPD_-limiting step. As the ∆*G*_RPD_-limiting steps and energies are evolutional with the change of descriptor value, the (*quasi*) activity and selectivity trend for CO_2_RR can be established, named as reaction phase diagram (RPD) analysis.

Compared to pristine Cu-N_4_, the boron dopants can effectively enhance the reactivity of Cu sites and the adsorption energies. For instance, the more stable COOH* and CHO* can promote the protonation of CO_2_ and CO, resulting in promoted production of CH_4_ over Cu-N_*x*_B_*y*_ sites at various applied potential (Fig. 1b and Supplementary Fig. 4). Projected density of states (PDOS) of CHO* adsorbed on different Cu-N_*x*_B_*y*_ sites explain well more stable adsorption energies (Fig. 1c–g and Supplementary Fig. 5). Different from the localized 2*p*-C states of adsorbed CHO* on Cu-N_4_ (Fig. 1c), it is more delocalized as the C atom (of CHO*) simultaneously binds with Cu and B at Cu-N_*x*_B_y_ sites, which indicates much stronger electronic interaction between CHO* and Cu-N_*x*_B_y_ sites. Besides, the hybridized peaks (i–x, as marked in Fig. 1d–g and Supplementary Fig. 5, respectively) below Fermi level show the strong electronic resonance between 2*p*-C (of CHO*) and 3*d*-Cu/2*p*-B of Cu-N_3_B, Cu-N_2_B_2_, Cu-NB_3_, and Cu-N_4_B_4_, suggesting enhanced reactivity on different Cu-N_*x*_B_y_ sites. This is also supported by analyzing the crystal orbital Hamilton population (COHP) (Supplementary Fig. 6). The COHP analysis showed that, the intermediate CHO* adsorbs on CuN_4_ through C atom bonding with Cu (C-Cu), where the antibonding states of C-Cu bond is partially occupied. With B substituting in Cu-N_4_ motif, the C-B bonds are much stronger than C-Cu, indicating the enhancement of CHO* adsorption at Cu-N_*x*_B_*y*_ sites. Besides, the marked peaks below Fermi level (**b-e**) refer to the strong electronic resonance between 2*p*-C (of CHO*) and 2*p*-B of Cu-N_*x*_B_*y*_ sites. Both PDOS and COHP analysis indicated that B substituting in Cu-N_4_ motif can promote the adsorption of key intermediates, providing an opportunity to boost the intrinsic activity of CO_2_-to-CH_4_.

### Synthesis and characterization of Cu single atom catalysts

**Fig. 2:**
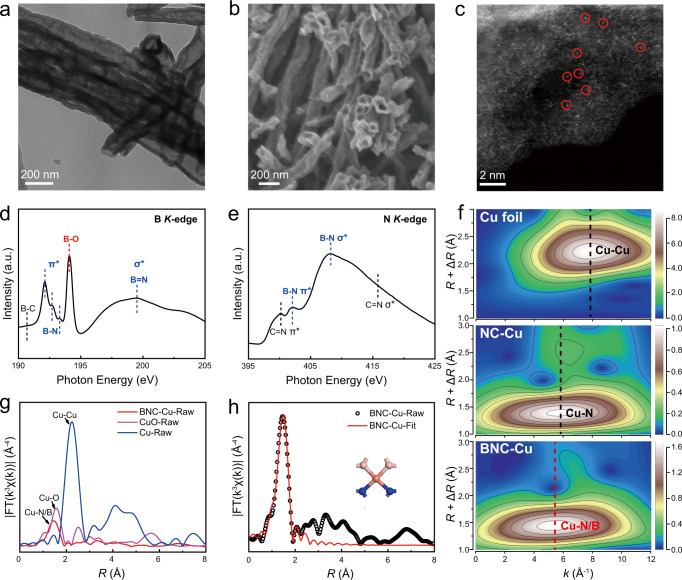
Characterization of BNC-Cu. **a**–**c** TEM image (**a**), SEM image (**b**), and HAADF-STEM image (**c**) of BNC-Cu. **d**, **e** B *K*-edge (**d**), N *K*-edge (**e**) XANES spectra of BNC-Cu. The B *K*-edge XANES for BNC-Cu showed the spectral fingerprints of B (sp^2^)-N (sp^2^) bonds with three B 1 *s* → π* resonances and one 1 *s* → σ* resonance. The resonance of B-C bonds could also be seen. The generally-existed interaction between B and N in BNC-Cu was further identified by signals of N 1 *s* → π* resonance and N 1 *s* → σ* resonance of B-N bonds in N *K*-edge XANES results, π* and σ* resonances of C = N were also observed. **f** WT-EXAFS plots of Cu foil, NC-Cu, and BNC-Cu. **g** FT-EXAFS spectra at Cu *K*-edge of BNC-Cu, CuO, and Cu foil. **h**, The corresponding EXAFS fitting curves of BNC-Cu at *R* space. The inset of **h** is the schematic model of the fitting result, Cu (orange), N (blue), B (pink).

Encouraged by these promising theoretical findings, we sought to construct an isolated Cu catalyst in which boron and nitrogen atoms are coordinated with Cu center. The lack of molecules containing Cu-N and Cu-B bond simultaneously limited the ball milling synthesis of a well-defined catalyst with a precise coordination configuration^43,44^. Hence, we managed to introduce B via a one-pot carbonization process (see details in “Methods”). Nevertheless, the high-temperature pyrolysis process may lead to the co-existence of several structures containing Cu-N_*x*_B_*y*_ motifs. We also prepared the Cu-N_4_ catalyst (NC-Cu) similarly but without boron precursor (Supplementary Figs. 7 and 8). Transmission electron microscopy (TEM) and scanning electron microscopy (SEM) images indicated that the BNC-Cu catalyst possessed a tubular structure with a mean external diameter of ~100 nm (Fig. 2a, b). X-ray diffraction (XRD) pattern and Raman spectrum only exhibited fingerprint peaks corresponding to graphitic carbon without Cu/CuO_*x*_/CuB_*x*_ signals, suggesting the formation of highly dispersed Cu species (Supplementary Figs. 9 and 10). The uniform distribution of Cu, B, C, and N was verified by energy-dispersive X-ray spectroscopy (EDS) elemental mapping (Supplementary Fig. 11), confirming the formation of B and N co-doped carbon support. The bright spots marked with red circles in high-angle annular dark-field scanning transmission electron microscopy (HAADF-STEM) image represented isolated Cu atoms dispersed in BNC support (Fig. 2c). Cu particles were not observed in HAADF-STEM images (Fig. 2c and Supplementary Fig. 11). Inductively coupled plasma atomic emission spectroscopy (ICP-AES) analysis revealed Cu loadings to be 2.5 wt.% for BNC-Cu and 2.7 wt.% for NC-Cu, respectively. X-ray photoelectron spectroscopy (XPS) results indicated the elemental composition of BNC-Cu with N: 17.2%, C: 38.0%, B: 16.9% (Supplementary Fig. 12a), demonstrating heavy doping of N and B into carbon matrix with similar doping-level. The high-resolution XPS spectrum for B 1 *s* (Supplementary Fig. 12b) suggested the existence of B-C, B-N, and B-O structure. N 1 *s* spectrum (Supplementary Fig. 12c) also displayed obvious component contribution corresponding to N-B^45–47^. The detailed information for high-resolution XPS spectra of NC-Cu could be found in Supplementary Fig. 13. Comparison between N 1 *s* spectra of BNC-Cu and NC-Cu (Supplementary Fig. 14) also indicated that different N species were generated from general interaction between B and N atoms.

We further employed X-ray absorption near-edge structure (XANES) spectroscopy to accurately identify the nitrogen and boron species in BNC-Cu. The B, N, and C *K*-edges XANES (Fig. 2d, e and Supplementary Fig. 15) for BNC-Cu exhibited the fingerprint peaks from B-C, B-N, and C-N, demonstrating the strong orbital interaction, via direct bonding, of arbitrary pairs from B, N, and C atoms^45,48,49^. Fourier transform infrared (FTIR) spectrum of BNC-Cu (Supplementary Fig. 16) also confirmed the existence of similar structures B-N, C-N, and B-C^50^. Meanwhile, the broadened N *K*-edge spectrum was associated with multiple forms of N species, corresponding well with XPS results. These results strongly supported that boron and nitrogen dopants were integrated into carbon matrix simultaneously, and interaction generally existed between such co-doped boron and nitrogen atoms. We then conducted XAS measurements (Fig. 2f–h and Supplementary Figs. 17–20) of Cu *K*-edge to reveal the geometric/electronic structure of isolated Cu center in BNC-Cu and NC-Cu. The Fourier transform extended X-ray absorption fine structure (FT-EXAFS) (Fig. 2g) of both samples showed only one main peak with similar radial distance at ~1.46 Å which was ascribed to Cu-N/Cu-B scattering path, different from typical Cu-O coordination at 1.57 Å in CuO. No characteristic peak of Cu-Cu contribution at 2.20 Å could be seen, confirming the atomic dispersion of Cu atoms on BNC support. To further investigate the first layer atomic coordination environment of Cu single atoms on BNC and NC support, we performed wavelet transform (WT) of the Cu *K*-edge EXAFS oscillations (Fig. 2f and Supplementary Fig. 19). The WT spectra for Cu foil and CuO showed intensity peaks at 7.90 and 6.40 Å^–1^, attributed to typical Cu-Cu and Cu-O coordination, respectively. However, for NC-Cu catalyst, the WT spectrum showed one dominated peak, with smaller *k* value than those of Cu-Cu and Cu-O coordination, indicating the sole formation of Cu-N bonds (Fig. 2f). We further observed the slight WT peak shift of BNC-Cu sample compared to NC-Cu, as shown in Fig. 2f, suggesting the subtle change of atomic coordination environment between BNC-Cu and NC-Cu. A positive shift of absorption edge in BNC-Cu compared with NC-Cu, as shown in Supplementary Fig. 17, indicated that the electronic structure of Cu atoms changed with the doping of B atoms. EELS point spectrum obtained at the edge of substrate (white box in Supplementary Fig. 21) on a small area, containing three Cu single atoms (bright dots), showed the colocation of B and N atoms around single Cu atoms within the carbon matrix. Considering the angstrom resolution of the electron probe, the signals in EELS point spectrum comes from the closest neighboring atoms of Cu single atoms^51,52^, suggesting a high possibility of Cu–N/B direct coordination in BNC-Cu^53–55^. Shift of XPS N 1 *s* spectrum of BNC-Cu compared with NC-Cu (Supplementary Fig. 13), due to the general interaction between B and N, also indicated the ubiquitous nearby boron atoms around nitrogen. Thus, the more positive Cu valence in BNC-Cu was very likely due to boron atoms that substituted nitrogen atoms and directly coordinated with Cu. Hence, taken above together, we could speculate that boron has been incorporated into the first coordination shell of isolated Cu, forming Cu-N_*x*_B_*y*_ motif. Furthermore, we fitted the EXAFS spectra of BNC-Cu and NC-Cu to illustrate the most possible local configuration of isolated Cu center, as shown in Fig. 2h, Supplementary Fig. 20, and Supplementary Tables 1 and 2. The best direct fitting results of FT-EXAFS showed that Cu center in NC-Cu mainly possessed Cu-N_4_ configuration, while the coordination numbers of Cu-N and Cu-B in BNC-Cu were 2.1 and 2.2, respectively, suggesting that the atomically dispersed Cu sites in BNC-Cu were most likely coordinated with two nitrogen atoms and two boron atoms. Considering that the resolved coordination numbers were not exactly 2, which was very likely due to both fitting error and the existence of other coordination structures in low content, we further conducted an inverse analysis by simulating the XANES spectra of different Cu-N_*x*_B_*y*_ centers with each optimized DFT models. The simulation result for Cu-N_2_B_2_ model showed the best alignment with experimental curve of BNC-Cu towards the main observed features, while the subtle mismatch of experimental spectrum and simulation result was probably ascribed to other Cu-N_*x*_B_*y*_ centers with a low proportion. Though we could not completely exclude the formation of other Cu-N_*x*_B_*y*_ centers, determined by the thermodynamic distribution, especially under a rapid and harsh pyrolysis condition, calculations of the formation energy among different coordination structures confirmed Cu-N_2_B_2_ as the most stable structure in BNC-Cu (Supplementary Fig. 22), again validating the majority of Cu-N_2_B_2_ center, consistent with the XAS analysis.

### Catalytic performance for CO_2_RR

**Fig. 3:**
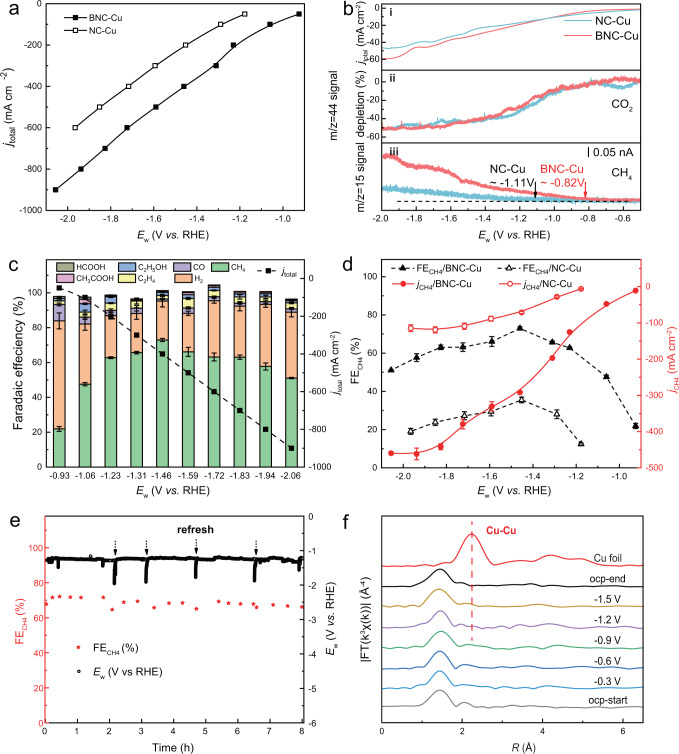
CO_2_RR performance of BNC-Cu and NC-Cu. **a** Total current density of BNC-Cu and NC-Cu at different applied potentials. **b** In situ DEMS data of BNC-Cu and NC-Cu. The total current densities (**i**), CO_2_ ionic currents (*m*/*z* = 44) (**ii**), and CH_4_ ionic currents (*m*/*z* = 15) (**iii**) for BNC-Cu and NC-Cu electrodes in CO_2_-saturated 0.5 M KHCO_3_ as measured in the negatively going potential sweep from −0.5 to −2.0 V vs. RHE at 1 mV s^−1^. **c** Total current densities and faradaic efficiencies for various products of BNC-Cu at different applied potentials, the error bars of FEs are calculated based on three independent measurements. **d** Faradaic efficiencies and partial current densities for CH_4_ of BNC-Cu and NC-Cu as a function of cathodic potentials. Red curve for partial current density and black curve for methane FE. The error bars of FE_CH4_ and *j*_CH4_ are calculated based on three independent measurements. **e** Stability test of CO_2_ to CH_4_ during an 8-h electrolysis under the current density of −200 mA cm^−2^. Red star for methane FE and black line for potential curve. **f** FT-EXAFS spectra of BNC-Cu at Cu *K*-edge under different applied potentials.

The electrocatalytic properties of BNC-Cu and NC-Cu towards CO_2_RR were measured in a flow reactor where 0.5 M KHCO_3_ aqueous solution was used as electrolyte (see details in “Methods”). We performed bulk electrolysis over BNC-Cu under different current density with potential range of -0.93 to −2.06 V vs. RHE (Fig. 3a) and over NC-Cu with potential range of −1.18 to −1.96 V vs. RHE (Supplementary Fig. 25). Gaseous and aqueous products were quantified by gas chromatography (GC) and ^1^H Nuclear Magnetic Resonance (NMR) (Supplementary Figs. 26 and 27), respectively. We found that, compared with NC-Cu, BNC-Cu exhibited much increased CO_2_RR current density under similar applied potentials. In addition, B-doped Cu-N_*x*_ active site in BNC-Cu demonstrated significant lower overpotential for CO_2_-to-CH_4_ conversion compared to Cu-N_4_ by in situ differential electrochemical mass spectrometry (DEMS) analysis (Fig. 3b and Supplementary Fig. 28). More specifically, defining the potential, where S/N of m/z = 15 signal is 5, as the onset potential for CH_4_ production, we could extrapolate the CH_4_ onset potential of −1.11 V vs. RHE for NC-Cu whereas that of BNC-Cu was −0.82 V vs. RHE (Supplementary Fig. 29). Such difference indicated that introduction of boron ligand into Cu-N_4_ motif did further promote its intrinsic activity for CO_2_-to-CH_4_.

For BNC-Cu, the hydrogen evolution reaction (HER) firstly dominated the cathodic reaction under low applied potentials, and then retreated dramatically with increased bias while CO_2_RR became the main cathodic reaction (Fig. 3c). The FE_CH4_ of BNC-Cu kept above 60% at a wide potential range from −1.23 to −1.83 V vs. RHE. The highest FE_CH4_ of 73% reached at −1.46 V vs. RHE with a *j*_CH4_ of −292 mA cm^–2^, as shown in Fig. 3d. The *j*_CH4_ continuously grew with increasing cathodic potential, reaching to an impressive value of −462 mA cm^–2^ at −1.94 V vs. RHE. In stark contrast, NC-Cu showed very mediocre performance for CH_4_ production. The FE for CH_4_ rarely surpassed 30% at tested potential range while the FE for H_2_ basically kept above 60% (Supplementary Fig. 25). Comparison of FE_H2_ and FE_CH4_ between such two catalysts at various similar potentials also confirmed the boost in CH_4_ production with BNC-Cu (Supplementary Figs. 30 and 31). H_2_-free selectivity of CH_4_ in CO_2_RR products under various potentials further confirmed the preferential production of CH_4_ over BNC-Cu (Supplementary Fig. 32). The contribution of substrates to CH_4_ production was rationally excluded, with both BNC and NC showing dominant HER activity and negligible CH_4_ production (Supplementary Figs. 33 and 34). Stability test of BNC-Cu manifested that the FE for CH_4_ maintained around 70% after 8-hour’s continuous electrolysis at −200 mA cm^–2^, without obvious potential fluctuation (Fig. 3e). Post-analysis of the cycled catalyst further demonstrated the structural stability of the formed Cu-N_*x*_B_*y*_ geometry for CO_2_-to-CH_4_ (Supplementary Fig. 20 and Supplementary Table 1). For the concern of intensely dynamic aggregation of atomic-dispersed Cu species to clusters during electrolysis, as proposed and demonstrated by other researchers^56,57^, in situ XAS experiments for BNC-Cu and NC-Cu at Cu *K*-edge under different applied potentials were also carried out. No visible signal of Cu-Cu coordination could be seen in both catalysts when applied with different negative potentials ranging from −0.3 to −1.5 V vs. RHE (Fig. 3f and Supplementary Fig. 35), indicating that the atomically dispersed Cu atoms remained as dominant Cu species during electrolysis. The above results revealed that BNC-Cu catalyst performed much better than the boron-free counterpart NC-Cu for converting CO_2_ to CH_4,_ validating the theoretical prediction results.

### Insight into CO_2_-to-CH_4_ conversion mechanism

**Fig. 4:**
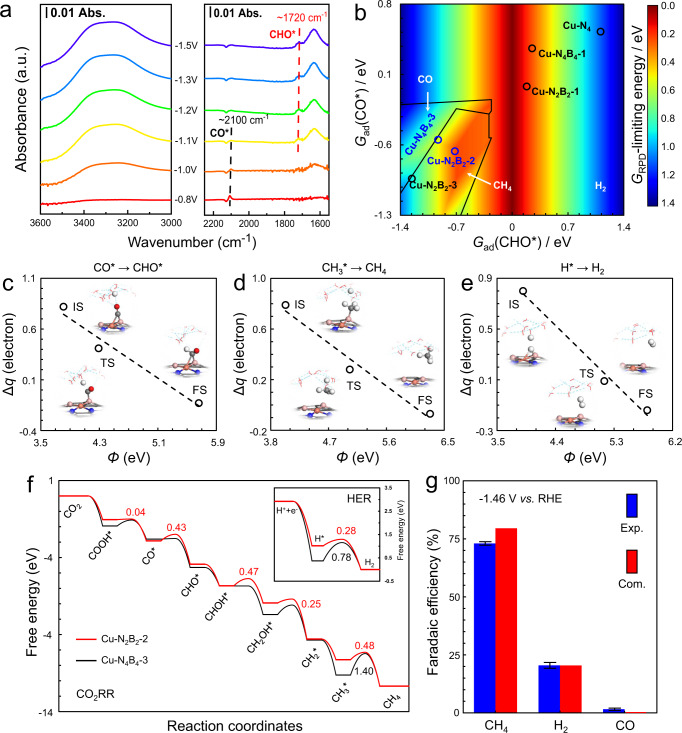
In situ ATR-IR and DFT calculations. **a** In situ ATR-IR spectra for BNC-Cu. **b** Two dimensional (*quasi*) activity and selectivity map for CO_2_RR and HER at −1.46 V vs. RHE, shown with two independent descriptors: *G*_ad_(CHO*) and *G*_ad_(CO*). **c**–**e** Calculated charge transfer (Δ*q*) and *Φ* on electrochemical interface at the initial states (IS), transition states (TS), and final states (FS) for CO* + H_2_O → CHO* + (OH^−^e^−^) (**c**), CH_3_* protonation (**d**), and Heyrovsky steps (**e**) over Cu-N_2_B_2_-2, while others are shown in Supplementary Figs. 37 and 38. **f** Reaction free energy diagram at −1.46 V vs. RHE for CO_2_RR and HER (inset) over Cu-N_2_B_2_-2 and Cu-N_4_B_4_-3 sites. **g** Comparison between computational and experimental Faradaic efficiencies at −1.46 V vs. RHE.

To further elucidate the CO_2_RR to CH_4_ mechanism over BNC-Cu, in situ attenuated total reflectance surface-enhanced infrared absorption spectroscopy (ATR-SEIRAS) measurements were conducted for BNC-Cu, as well as the NC-Cu control (Fig. 4a and Supplementary Fig. 36). As depicted in Fig. 4a, on applying potential from −0.8 to −1.5 V vs. RHE over BNC-Cu, two peaks could be well noted. Both peaks showed a slight red-shift with increasing applied potential, indicating that such observed signals originated from the in situ formed CO_2_RR intermediates^58^. We ascribed the detected two peaks at around 2100 and 1720 cm^–1^ to CO*^59,60^ and the key intermediate to produce CH_4_^21–23,61^, CHO*^62^, respectively. On scanning to a negative potential, with the gradual disappearance of CO* signal, we observed the accumulation of CHO* intermediate, revealing the rapid transformation of CO* into CHO* at high applied overpotential. On the other hand, no visible CHO* peak was observed for NC-Cu (Supplementary Fig. 36), while only the CO* fingerprint peak appeared and the intensity remained almost constant over the tested potential range, revealing higher barrier for CO* hydrogenation to CHO* on NC-Cu. Such a difference on CO*-to-CHO* transition may explain the CO_2_RR performance gap between BNC-Cu and NC-Cu. Besides, the ATR-SEIRAS displayed another two strong broad peaks at around 1650 and 3400 cm^–1^, which could be ascribed to the interfacial H_2_O^63,64^. We noted the intensity of these peaks increased with increasing applied potential, suggesting that water molecules tended to adsorb on the surface of BNC-Cu during CO_2_RR (Fig. 3a) ^65^. Such readily adsorbed water molecules could provide adequate hydrogen source for CH_4_ production, but this trend was not obvious for NC-Cu (Supplementary Fig. 36).

The observed signals of CHO* further corroborated the use of CHO* as the target in the aforementioned computational screening over a series of Cu-N_*x*_B_*y*_ sites (Supplementary Figs. 2 and 3). Based on the resolving results of Cu center in BNC-Cu, it is reasonable to speculate the boost of CH_4_ production was very likely due to the dominant Cu-N_2_B_2_ structure, and we thus performed further theoretical investigations to understand such high selectivity towards CH_4_ with barrier calculations in consideration of several structures containing Cu-N_2_B_2_ motif (Cu-N_2_B_2_−1, Cu-N_2_B_2_−2, Cu-N_2_B_2_−3, Cu-N_4_B_4_−1, and Cu-N_4_B_4_−3, illustrated in Supplementary Figs. 2 and 3). On the basis of one dimensional (*quasi*) activity map using *G*_ad_(CHO*) as the primary descriptor (Fig. 1b), we chose *G*_ad_(CO*) as the second descriptor as the adsorption strength of CO* is tightly correlated with CO production, thus forming a two-dimensional (*quasi*) activity and selectivity map that clearly shows the selectivity trend of Cu-N_4_, Cu-N_2_B_2_, and Cu-N_4_B_4_ sites (Fig. 4b). At −1.46 V vs. RHE, the maximum FE_CH4_ of BNC-Cu observed in experiments, HER is thermodynamically favorable at sites with weak surface reactivity, for instance, Cu-N_4_ site. It again demonstrates how corporation of B atoms into Cu-N_4_ structure augments CO_2_RR to CH_4_. However, CO_2_RR is the main reaction on sites with strong adsorption of intermediates, where relatively weak CO* adsorption is prone to desorb and produce CO, otherwise to CH_4_ production. Accordingly, the site Cu-N_2_B_2_−2, as well as Cu-N_4_B_4_−3, are more likely to contribute in CO_2_ to CH_4_ process among other structures.

To further make comparison between Cu-N_2_B_2_−2 and Cu-N_4_B_4_−3, electrochemical barriers of CO_2_RR and HER over the two structures were calculated and shown in Fig. 4c–f. The electrochemical barriers of proton-coupled electron transfer reactions were calculated via a capacitor model^66,67^. According to “charge-extrapolation” method^67,68^, the amount of electron transfer (Δ*q*) from water to the electrode surface is linearly correlated with the relative work function (*Φ*) at the initial states (IS), transition states (TS), and final states (FS). Figure 4c–e show such linear correlations of the critical steps for products selectivity. Illustrated by Fig. 4c, the TS of CO* protonation step to CHO* is IS-like, giving rise to a small charge transfer coefficient (β). A median TS of the ∆*G*_RPD_-limiting step for CO_2_RR, CH_3_* protonation (Fig. 4d), leads to a moderate β. However, the β of Heyrovsky step, the ∆*G*_RPD_-limiting step for HER, is larger due to its FS-like TS (Fig. 4e). It accurately predicts the high FE of H_2_ at very negative potentials. Based on calculated kinetic barriers, a detailed free energy landscape of CO_2_RR over Cu-N_2_B_2_−2 and Cu-N_4_B_4_−3 sites is shown in Fig. 4f (HER in subfigure). The CO* protonation has a barrier of 0.43 eV on Cu-N_2_B_2_−2, while such process is barrier-less on Cu-N_4_B_4_−3. However, for CH_3_* protonation, the larger barrier on Cu-N_4_B_4_−3 of 1.40 eV than that on Cu-N_2_B_2_−2 of 0.48 eV supports well the significance of global energy optimization. Specifically, Cu-N_2_B_2_−2 shows lower barriers of the most energetically difficult steps than Cu-N_4_B_4_−3 for CO_2_RR, which is consistent with the (*quasi*) activity map in Fig. 4b. Thus, Cu-N_2_B_2_−2 site is expected to show prominent CH_4_ production during CO_2_RR. The following microkinetic modeling was conducted for Cu-N_2_B_2_−2 at −1.46 V vs. RHE (Fig. 4g). The similarity of selectivity between computational and experimental data inversely suggested that Cu-N_2_B_2_−2 sites are probably the dominant sites among various possible Cu-containing sites. The experimental FE_CH4_ value was 75%, which was lower than the simulated result of 80%. This discrepancy was due to the existence of other sites that were present in small proportions and did not have as high a methane productivity. The comparable product distribution also inversely validated our speculation that the majority of Cu-containing sites was Cu-N_2_B_2_, resolved by multiple XAS-relative studies. Compared with the pristine Cu-N_4_, the enhanced surface reactivity and appropriate adsorption energies over Cu-N_2_B_2_−2 is thus proposed as the chemical origin of the high selectivity towards CH_4_ for as-synthesized BNC-Cu catalyst.

In summary, by virtue of thermodynamic trend and global energy optimization analysis, refined manipulation of experimental synthesis and characterizations, theoretical microkinetic modeling, this work showcased a general way to design Cu single atom catalysts towards methane production via rationally regulating the nearest coordination environment. By modifying the Cu-N_4_ sites with partial B substitution, the enhanced adsorption of CO* and CHO* intermediates are theoretically proven to be beneficial for CH_4_ generation. Experimentally, as-obtained B-doped Cu-N_*x*_ active sites exhibited a high CH_4_ FE of 73% at −1.46 V vs. RHE and a maximal *j*_CH4_ of −462 mA cm^–2^ at −1.94 V vs. RHE, respectively. This work implicates a valuable avenue for bolstering selectivity of Cu towards a specific product.

## Methods

### Computational details

All density functional theory (DFT) calculations in this work were performed via the Vienna Ab-initio Simulation Package (VASP)^69,70^. The generalized gradient approximation (GGA)^71^ with the revised Perdew–Burke–Ernzerhof (rPBE) functional^72^ (GGA-rPBE) was employed to describe the electron interactions. The projected augmented wave (PAW)^73,74^ was employed to describe the valence electrons with a plane wave basis sets and the kinetic energy cutoff was set to 400 eV. Structural optimizations were performed with the residual force and electronic energy differences smaller than −0.05 eV Å^–1^ and 10^–5 ^eV, respectively. In addition, dispersion effects have been taken into account by DFT-D3 method. Locating transition states were conducted by the climbing image nudged elastic band (CI-NEB)^75^ and dimer^76,77^ methods, where the convergence force was set as smaller than 0.1 eV Å^−1^. Moreover, a Gaussian smearing with a width of 0.2 eV and a Monkhorst–Pack *k*-point mesh grid of 2 × 2 × 1 were used. For atomic model construction, Cu-N_4_ was embedded in a graphene structure of 17.16 × 14.87 Å^2^ as the active center of NC-Cu (Supplementary Fig. 1), where a vacuum of 15 Å was introduced to avoid the interaction of adjacent layers. Several typical structures with different amounts of boron incorporated were also optimized (Supplementary Fig. 1).

The adsorption energy (*E*_ad_) of intermediates refers to the energies of gas-phase CO(g), H_2_(g), and H_2_O(g), the chemical potential of single H, O, C atom is ${E}_{{{{{{\rm{H}}}}}}}=\frac{1}{2}{E}_{{{{{{{\rm{H}}}}}}}_{2}}$, ${E}_{{{{{{\rm{O}}}}}}}={E}_{{{{{{{\rm{H}}}}}}}_{2}{{{{{\rm{O}}}}}}}-{E}_{{{{{{{\rm{H}}}}}}}_{2}}$, and ${E}_{{{{{{\rm{C}}}}}}}={E}_{{{{{{{\rm{CO}}}}}}}}-{E}_{{{{{{\rm{O}}}}}}}$. The intermediates adsorption energy (*E*_ad_) can be computed by the following equation:

$${E}_{{{{{{{\rm{ad}}}}}}}}={E}_{{{{{{{\rm{tot}}}}}}}}-{E}_{{{{{{{\rm{bare}}}}}}}}-({\alpha E}_{{{{{{\rm{C}}}}}}}+{\beta E}_{{{{{{\rm{H}}}}}}}+\gamma {E}_{{{{{{\rm{O}}}}}}})$$

where *E*_bare_ and *E*_tot_ are the energies of bare catalysts and the catalysts with adsorbates, respectively. The numbers of carbon, hydrogen and oxygen atoms in intermediates are described by coefficients *α*, *β*, and *γ*. Furthermore, free energy corrections were conducted in this work at the temperature of 298 K, with the scheme described in the previous work^78^.

The formation energy of CuN_*x*_B_4-*x*_ sites (*x* = 0–3) are computed via the following equation:

$${E}_{{{{{{\rm{f}}}}}}}={E}_{{{{{{{\rm{total}}}}}}}}-\sum {n}_{i}{\mu }_{i}$$

where *n*_*i*_ and *μ*_*i*_ refer to the number and chemical potential of element *i*, respectively. The energies of Cu and C are referenced to the Cu bulk and graphene, respectively. Besides, the chemical potentials of N and B are calculated via the energies of HNO_3_, H_3_BO_3_, NH_2_CN, and H_2_O.

### The elementary steps of electrochemical CO_2_RR and HER

We have considered three whole reactions:

$${{{{{{\rm{CO}}}}}}}_{2}+6{{{{{{\rm{H}}}}}}}_{2}{{{{{\rm{O}}}}}}\to {{{{{{\rm{CH}}}}}}}_{4}+8({{{{{{\rm{OH}}}}}}}^{-}-{{{{{{\rm{e}}}}}}}^{-})$$

$${{{{{{\rm{CO}}}}}}}_{2}+{{{{{{\rm{H}}}}}}}_{2}{{{{{\rm{O}}}}}}\to {{{{{\rm{CO}}}}}}+2({{{{{{\rm{OH}}}}}}}^{-}-{{{{{{\rm{e}}}}}}}^{-})$$

$$2{{{{{{\rm{H}}}}}}}_{2}{{{{{\rm{O}}}}}}\to {{{{{{\rm{H}}}}}}}_{2}+2({{{{{{\rm{OH}}}}}}}^{-}-{{{{{{\rm{e}}}}}}}^{-})$$

In which several elementary reactions may be involved, as listed in the following:

R1 $${{{{{{\rm{CO}}}}}}}_{2}+{{{{{{\rm{H}}}}}}}_{2}{{{{{\rm{O}}}}}}+\;{^*} \to {{{{{\rm{COOH}}}}}}^ \ast+({{{{{{\rm{OH}}}}}}}^{-}-{{{{{{\rm{e}}}}}}}^{-})$$

R2 $${{{{{\rm{COOH}}}}}}^ \ast+{{{{{{\rm{H}}}}}}}_{2}{{{{{\rm{O}}}}}}\to {{{{{\rm{HOCOH}}}}}}^ \ast+({{{{{{\rm{OH}}}}}}}^{-}-{{{{{{\rm{e}}}}}}}^{-})$$

R3 $${{{{{{\rm{HOCOH}}}}}}}^*\to {{{{{\rm{COH}}}}}}^ \ast+({{{{{{\rm{OH}}}}}}}^{-}-{{{{{{\rm{e}}}}}}}^{-})$$

R4 $${{{{{\rm{COOH}}}}}}^*\to {{{{{\rm{CO}}}}}}^ \ast+({{{{{{\rm{OH}}}}}}}^{-}-{{{{{{\rm{e}}}}}}}^{-})$$

R5 $${{{{{\rm{CO}}}}}}^ \ast+{{{{{{\rm{H}}}}}}}_{2}{{{{{\rm{O}}}}}}\to {{{{{\rm{COH}}}}}}^ \ast+({{{{{{\rm{OH}}}}}}}^{-}-{{{{{{\rm{e}}}}}}}^{-})$$

R6 $${{{{{\rm{CHO}}}}}}^ \ast+{{{{{{\rm{H}}}}}}}_{2}{{{{{\rm{O}}}}}}\to {{{{{\rm{COH}}}}}}^ \ast+({{{{{{\rm{OH}}}}}}}^{-}-{{{{{{\rm{e}}}}}}}^{-})$$

R7 $${{{{{{\rm{CH}}}}}}}_{2}{{{{{\rm{O}}}}}}^ \ast+{{{{{{\rm{H}}}}}}}_{2}{{{{{\rm{O}}}}}}\to {{{{{{\rm{CH}}}}}}}_{3}{{{{{\rm{O}}}}}}^ \ast+({{{{{{\rm{OH}}}}}}}^{-}-{{{{{{\rm{e}}}}}}}^{-})$$

R8 $${{{{{{\rm{CH}}}}}}}_{2}{{{{{\rm{O}}}}}}^ \ast+{{{{{{\rm{H}}}}}}}_{2}{{{{{\rm{O}}}}}}\to {{{{{\rm{CHOH}}}}}}^ \ast+({{{{{{\rm{OH}}}}}}}^{-}-{{{{{{\rm{e}}}}}}}^{-})$$

R9 $${{{{{\rm{CHOH}}}}}}^ \ast+{{{{{{\rm{H}}}}}}}_{2}{{{{{\rm{O}}}}}}\to {{{{{{\rm{CH}}}}}}}_{2}{{{{{\rm{OH}}}}}}^ \ast+({{{{{{\rm{OH}}}}}}}^{-}-{{{{{{\rm{e}}}}}}}^{-})$$

R10 $${{{{{{\rm{CH}}}}}}}_{2}{{{{{\rm{O}}}}}}^ \ast+{{{{{{\rm{H}}}}}}}_{2}{{{{{\rm{O}}}}}}\to {{{{{{\rm{CH}}}}}}}_{2}{{{{{\rm{OH}}}}}}^ \ast+({{{{{{\rm{OH}}}}}}}^{-}-{{{{{{\rm{e}}}}}}}^{-})$$

R11 $${{{{{\rm{CO}}}}}}^ \ast+{{{{{{\rm{H}}}}}}}_{2}{{{{{\rm{O}}}}}}\to {{{{{\rm{COH}}}}}}^ \ast+({{{{{{\rm{OH}}}}}}}^{-}-{{{{{{\rm{e}}}}}}}^{-})$$

R12 $${{{{{\rm{COH}}}}}}^ \ast+{{{{{{\rm{H}}}}}}}_{2}{{{{{\rm{O}}}}}}\to {{{{{\rm{CHOH}}}}}}^ \ast+({{{{{{\rm{OH}}}}}}}^{-}-{{{{{{\rm{e}}}}}}}^{-})$$

R13 $${{{{{{\rm{CH}}}}}}}_{3}{{{{{\rm{O}}}}}}^ \ast+{{{{{{\rm{H}}}}}}}_{2}{{{{{\rm{O}}}}}}\to {{{{{{\rm{CH}}}}}}}_{4}+{{{{{\rm{O}}}}}}^ \ast+({{{{{{\rm{OH}}}}}}}^{-}-{{{{{{\rm{e}}}}}}}^{-})$$

R14 $${{{{{{\rm{CH}}}}}}}_{2}{{{{{\rm{OH}}}}}}^*\to {{{{{{{\rm{CH}}}}}}}_{2}}^ \ast+({{{{{{\rm{OH}}}}}}}^{-}-{{{{{{\rm{e}}}}}}}^{-})$$

R15 $${{{{{{{\rm{CH}}}}}}}_{2}}^ \ast+{{{{{{\rm{H}}}}}}}_{2}{{{{{\rm{O}}}}}}\to {{{{{{{\rm{CH}}}}}}}_{3}}^ \ast+({{{{{{\rm{OH}}}}}}}^{-}-{{{{{{\rm{e}}}}}}}^{-})$$

R16 $${{{{{{{\rm{CH}}}}}}}_{3}}^ \ast+{{{{{{\rm{H}}}}}}}_{2}{{{{{\rm{O}}}}}}\to {{{{{{\rm{CH}}}}}}}_{4}+\;^{*}+({{{{{{\rm{OH}}}}}}}^{-}-{{{{{{\rm{e}}}}}}}^{-})$$

R17 $${{{{{\rm{O}}}}}}^ \ast+{{{{{{\rm{H}}}}}}}_{2}{{{{{\rm{O}}}}}}\to {{{{{\rm{OH}}}}}}^ \ast+({{{{{{\rm{OH}}}}}}}^{-}-{{{{{{\rm{e}}}}}}}^{-})$$

R18 $${{{{{\rm{OH}}}}}}^*\to ({{{{{{\rm{OH}}}}}}}^{-}-{{{{{{\rm{e}}}}}}}^{-})+\;^{*}$$

R19 $${{{{{\rm{CO}}}}}}^*\to {{{{{\rm{CO}}}}}}+\;^{*}$$

R20 $${{{{{{\rm{H}}}}}}}_{2}{{{{{\rm{O}}}}}}+\;^{*} \to {{{{{\rm{H}}}}}}^ \ast+({{{{{{\rm{OH}}}}}}}^{-}-{{{{{{\rm{e}}}}}}}^{-})$$

R21 $${{{{{\rm{H}}}}}}^ \ast+{{{{{{\rm{H}}}}}}}_{2}{{{{{\rm{O}}}}}}\to {{{{{{\rm{H}}}}}}}_{2}+\;^{*}+({{{{{{\rm{OH}}}}}}}^{-}-{{{{{{\rm{e}}}}}}}^{-})$$

R22 $$2{{{{{\rm{H}}}}}}^*\to {{{{{{\rm{H}}}}}}}_{2}+2^*$$

### Theoretical XANES spectrum calculations

The Cu *K*-edge XANES simulation was conducted with the FDMNES code in multiple scattering mode (Green) using the muffin-tin potential. The energy dependent exchange-correlation potential was calculated in the real Hedin–Lundqvist scheme, and then the spectra convoluted using a Lorentzian function with an energy-dependent width to account for the broadening due both to the core-hole width and to the final state width^79,80^. Parameter optimization was performed by comparing the theoretical and experimental spectra to acquire the most appropriate convolution parameters. The calculated models were built based on DFT calculations to avoid manual bias.

### Chemicals

All chemicals were used as received without further purification. Copper (II) nitrite trihydrate (Cu(NO_3_)_2_·3H_2_O, AR, 99%), cyanamide (NCNH_2_), boric acid (H_3_BO_3_, GR, 99.8%) and polyethylene glycol-2000 (PEG-2000, average M_n_ = 2000) were purchased from Shanghai Macklin Biochemical Co., Ltd (Shanghai, China). Sustainion® XA-9 Alkaline Ionomer (5 % in Ethanol) was purchased from Dioxide Materials^TM^. All the chemicals were used without further purification.

### Materials synthesis

BNC-Cu was synthesized through a one-pot carbonization process, copper nitride, boric acid, cyanamide, PEG-2000 acting as copper, boron, nitrogen and carbon source and soft-template respectively. In a typical synthesis, copper (II) nitrite trihydrate (0.15 g), boric acid (0.6 g), cyanamide (14.0 g), and PEG-2000 (2.0 g) were dissolved in 120 mL of deionized water under ultrasonication for 15 min. The homogeneous solution was then heated to 120 °C for 12 h under reflux and continued stir. A gray crystalline powder was obtained upon rotary evaporation of the solvent. The obtained solid mixture was annealed at 900 °C for 6 h with a heating rate of 5 °C min^−1^ under the protection of argon, and the obtained final product was BNC-Cu. The NC-Cu catalyst was synthesized similarly with 0.1 g copper (II) nitrite trihydrate input and without boric acid. The pure-BNC substrate was also synthesized without copper precursor, while pure NC substrate was synthesized without both coppern precursor and boric acid.

### Characterization techniques

The as-synthesized BNC-Cu and NC-Cu were characterized by various analytical techniques. X-ray diffraction (XRD) was performed on a Philips X’Pert Pro Super diffractometer with Cu-*K*α radiation (*λ*  =  1.54178 Å). The morphology of the samples was observed by scanning electron microscopy (SEM, Zersss Supra 40) and transmission electron microscopy (TEM, Hitachi H-7650). HAADF-STEM images, energy-dispersive X-ray Spectroscopy (EDS) elemental mapping, and electron energy loss spectroscopy (EELS) were carried out on JEOL ARM-200F field-emission transmission electron microscope operating at an accelerating voltage of 200 kV using Mo-based TEM grids. Raman spectra were taken on a Raman microscope (Renishaw®) excited with a 785 nm excitation laser. X-ray photoelectron spectroscopy (XPS) measurements were performed on a VG ESCALAB MK II X-ray photoelectron spectrometer with Mg *K*α  =  1253.6 eV as the exciting source. Soft X-ray absorption spectra (B *K*-edge, N *K*-edge, and C *K*-edge) were carried out at the Catalysis and Surface Science Endstation at the BL11U beamline in the National Synchrotron Radiation Laboratory (NSRL) in Hefei, China.

### Electrochemical measurements

The electrochemical measurements were conducted with an electrochemical workstation (CHI 660E, Shanghai CH Instruments). The Ag/AgCl wire in saturated KCl solution was adopted as the reference electrode, and the counter anodic reaction was oxygen evolution reaction over a Ni foam. All potentials were converted to the RHE reference scale using the relation *E*_RHE_ = *E*_Ag/AgCl_ + 0.197 + pH × 0.059. Solution resistance was determined by potentiostatic electrochemical impedance spectroscopy at frequencies ranging from 0.1 Hz to 200 kHz and compensated by 85%. To prepare a catalyst cathode in a flow reactor, the catalyst ink was prepared at first with a constant composition ratio for each sample, 10 mg of catalyst, mixed with 25 μL of ionomer in 2 mL of isopropanol. Such ink was then airbrushed onto a 2 cm × 1.5 cm carbon gas diffusion layer (39BC) under the heating of a heat stage set in 80 °C. The mass loading for each catalyst was determined by weighing the mass of carbon paper before and after sprayed with catalyst ink and controlled to be ~1.0 mg cm^−2^. Such two electrodes were then placed on opposite sides of two polytetrafluoroethylene (PTFE) sheets with 0.4 cm × 1.5 cm channels. The geometric surface area of catalysts was controlled as 0.6 cm^2^. A Nafion 115 membrane (Fuel Cell Store) was sandwiched between the two PTFE sheets to separate the chambers. On the cathode side, a titanium gas flow chamber supplied 30 s.c.c.m. CO_2_ (monitored by an Alicat Scientific mass flow controller). 0.5 M KHCO_3_ electrolyte was pumped through cathode chambers with a constant rate of 0.75 mL min^−1^, while 1 M KOH served as anodic electrolyte was circulated around the anode with a rate of 25 mL min^−1^.

### CO_2_ reduction product analysis

To quantify the gas products obtained during CO_2_ electrolysis, pure CO_2_ gas was delivered into the cathodic compartment at a constant rate and vented into a gas chromatograph (PerkinElmer Clarus® 690) equipped with a thermal conductivity detector and a flame ionization detector. The liquid products were quantified using a 400 MHz NMR spectrometer. Typically, after electrolysis, 600 μL electrolyte was mixed with 100 μL D_2_O (Sigma Aldrich, 99.9 at.% D) and 0.05 μL dimethylsulfoxide (Sigma Aldrich, 99.9%) as internal standard.

### Ex situ and in situ XAS experiments

The ex situ X-ray absorption spectroscopy (XAS) spectra of Cu *K*-edge were obtained using beamline 44 A of Taiwan Photon Source (TPS) at National Synchrotron Radiation Research Center, Taiwan. All XAS data of Cu *K*-edge were collected in fluorescence mode using 7-element SDD detector and the incident photon energy were calibrated using standard Cu foil. In situ XAS spectra of Cu K-edge were obtained using beamline BL11B and BL14W at the Shanghai Synchrotron Radiation Facility (SSRF), Shanghai advanced Research Institute, Chinese Academy of Sciences. All XAS data of Cu *K*-edge were collected in fluorescence mode and the incident photon energy were calibrated using standard Cu foil. A self-design organic glass electrochemical cell was set in a three-electrode configuration and employed for our in situ XAS experiments. A rectangular orgonic glass cap was used to cover the cell and to keep the cell at fixed position of optical path. Several holes on the cap are used for CO_2_ bubbling and ensuring a fixed distance between working and reference electrodes for all experiment. A graphite rod and an Ag/AgCl electrode were used as the counter electrode and reference electrode, respectively. The working cell has flat walls with a single circular hole of 2 cm in diameter as a window of contact between electrolyte and catalysts, and a beam of synchrotron radiation X-ray light irradiated within the circular area during the in situ XAS experiments. To prepare a catalyst working electrode, 18 mg of catalyst, mixed with 45 μL of ionomer in 4 mL of isopropanol, such ink was then airbrushed onto a 2.5 cm × 2.5 cm carbon gas diffusion layer (39BC) with a mass loading of ~1.0 mg cm^−2^. Catalyst coated carbon paper was in contact with a slip of copper with the catalyst layer facing inward. Then 20 mL 0.5 M KHCO_3_ solution pre-saturated with CO_2_ was poured into the cell. The solution was not stirred and CO_2_ was bubbled into the solution bottom through the hole on that orgonic glass cap during the experiment. The flow rate of CO_2_ was 10 s.c.c.m. monitored by an Alicat Scientific mass flow controller. The cell was connected to an electrochemical station by making electrical contact to the copper tape slip that protruded from the side of the working cell. Before the in situ XAS experiments, XAFS spectra were recorded at different positions on the electrode to check the homogeneity of the catalyst. During the in situ XAS experiments, the potential on working electrode started from ocp to a series of cathodic potentials, and back to ocp. At each potential, the system was allowed to equilibrate for 10 min before recording a spectrum, then scans at the Cu *K*-edge were recorded. Data reduction, data analysis, and EXAFS fitting for XAS analysis in this work were performed with the Athena, Artemis, and IFEFFIT software packages. For quantitative analysis, phase shifts and backscattering amplitudes were generated by the FEFF calculations based on crystal structures of Cu, and were then calibrated through performing the FEFFIT of the EXAFS data of the reference samples, mainly to obtain the amplitude reduction factor (*S*_0_^2^) values. With *S*_0_^2^ known, the EXAFS data of the catalyst materials were fitted with such generated phase shifts and amplitudes.

### In situ DEMS experiments

The “probe-type” DEMS was applied for the detection of volatile CH_4_ and H_2_ produced during the CO_2_RR, as well as the reactant CO_2_ consumed. The whole tests were conducted in a flow-cell system with a gas diffusion electrode as working electrode. To prepare a catalyst working electrode, 10 mg of catalyst, mixed with 25 μL of ionomer in 2 mL of isopropanol, such ink was then airbrushed onto a 2 cm × 1.5 cm carbon gas diffusion layer (39BC) with a mass loading of ~1.0 mg cm^−2^. Catalyst coated carbon paper was in contact with a slip of copper with the catalyst layer facing inward to the electrolyte. The effective area of the working electrode is a circle of 1 cm in diameter. A sampling probe approached the working electrode at a distance of ca. 20 μm, and a peristaltic pump replaced the solution near the working electrode at a flow rate of 1.25 mL min^–1^. The onset potential for each product is defined as the potential where the S/N of corresponding m/z signal is 5.

### In situ attenuated total reflection surface-enhanced IR absorption spectroscopy (ATR-SEIRAS) measurements

In situ ATR-SEIRAS spectrum was gathered by a FT-IR spectrometer (Thermo Scientific Nicolet iS50) equipped with MCT-A detector. The catalyst inks were prepared by mixing 10 mg electrocatalysts, 5 mL ethanol, and 25 μL of ionomer. 10 μL of ink solution was dropped onto the central area (confined by an O-ring with *Φ* = 8 mm) of an Au film deposited on the basal plane of a hemicylindrical Si prism by evaporation. The Si prism was assembled in a spectro-electrochemical cell with Pt wire as a counter electrode, Ag/AgCl wire in saturated KCl solution as reference electrode, and 0.5 M KHCO_3_ solution pre-saturated and continuously bubbled with CO_2_ as electrolyte. All spectra are collected at a resolution of 4 cm^−1^ and each single-beam spectrum is an average of 200 scans. A CHI 660e electrochemistry workstation (Shanghai CH Instruments, Inc.) was used for potential control.

## Supplementary information

Supplementary Information

## Data Availability

The data that support the findings of this study are available from the corresponding authors upon reasonable request. Source data are provided with this paper.

## Source data

Source Data
