# Supplementary material for: Manipulating local coordination of copper single atom catalyst enables efficient CO2-to-CH4 conversion
Source: Nat Commun. 2023 Jun 8;14:3382. doi: 10.1038/s41467-023-39048-6 (PMC10250324; doi:10.1038/s41467-023-39048-6)
Supplement: Supplementary file 1 — Supplementary Information [file 41467_2023_39048_MOESM1_ESM.pdf]

Supplementary Information for

Manipulating local coordination of copper single atom catalyst enables efficient CO_2-to-CH_4 conversion

Yizhou Dai[1,2]†, Huan Li[3,4]†, Chuanhao Wang[1,2]†, Weiqing Xue[1,2], Menglu Zhang[1,2], Donghao Zhao[1], Jing Xue[1,2], Jiawei Li[1,2], Laihao Luo[1,2], Chunxiao Liu[1,2], Xu Li[1], Peixin Cui[5], Qiu Jiang[2], Tingting Zheng[2], Songqi Gu[6], Yao Zhang[1], Jianping Xiao[3,4*], Chuan Xia[2,7*], Jie Zeng[1,8*]

[1]Hefei National Research Center for Physical Sciences at the Microscale, University of Science and Technology of China, Hefei, Anhui 230026, P. R. China.

[2]School of Materials and Energy, University of Electronic Science and Technology of China, Chengdu 611731, P. R. China.

[3]State Key Laboratory of Catalysis, Dalian Institute of Chemical Physics, Dalian National Laboratory for Clean Energy, Chinese Academy of Sciences, Dalian 116023, P. R. China.

[4]University of Chinese Academy of Sciences, Beijing 100049, P. R. China.

[5]Key Laboratory of Soil Environment and Pollution Remediation, Institute of Soil Science, Chinese Academy of Sciences, Nanjing 210008, P. R. China.

[6]Shanghai Advanced Research Institute, Chinese Academy of Sciences, Shanghai 201210, P. R. China

[7]Yangtze Delta Region Institute (Huzhou), University of Electronic Science and Technology of China, Huzhou, Zhejiang 313001, China.

[8]School of Chemistry & Chemical Engineering, Anhui University of Technology, Ma'anshan, Anhui 243002, P. R. China.

*Corresponding author: E-mail: xiao@dicp.ac.cn (J.X.); chuan.xia@uestc.edu.cn (C.X.); zengj@ustc.edu.cn (J.Z.);

†These authors contributed equally to this work.

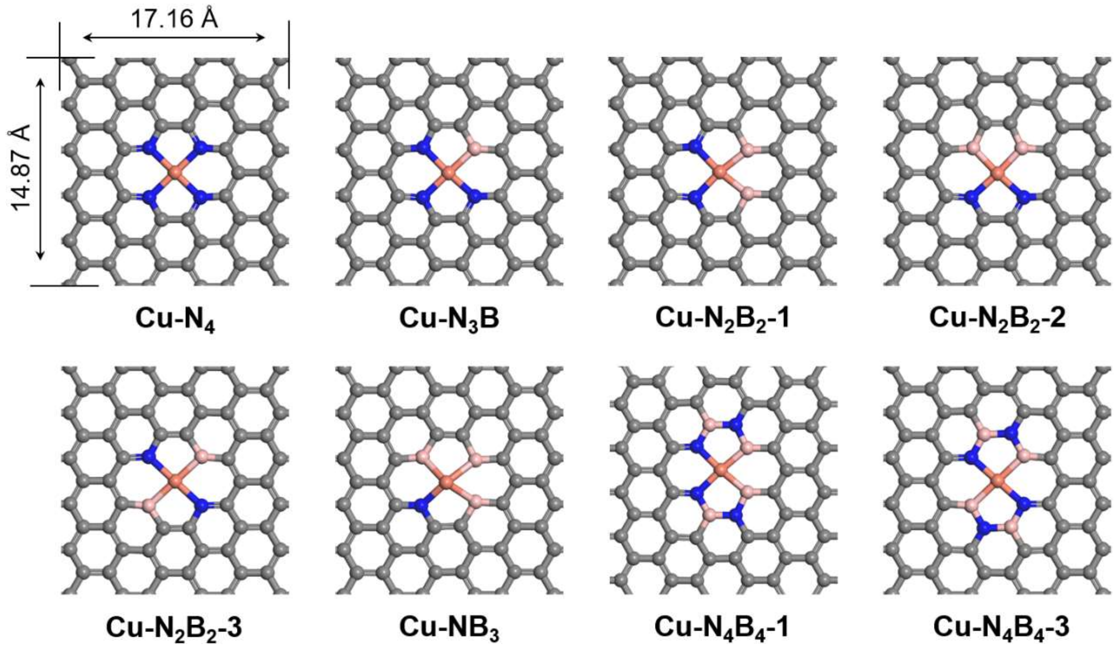

Supplementary Fig. 1 | The optimized structures of several typical structures with different boron concentrations (Cu-N_xB_y). Based on Cu-N_4 confined in carbon matrix, typical structures with different boron concentrations (Cu-N_xB_y) were optimized by directly substituting several N or C atoms with B atoms.

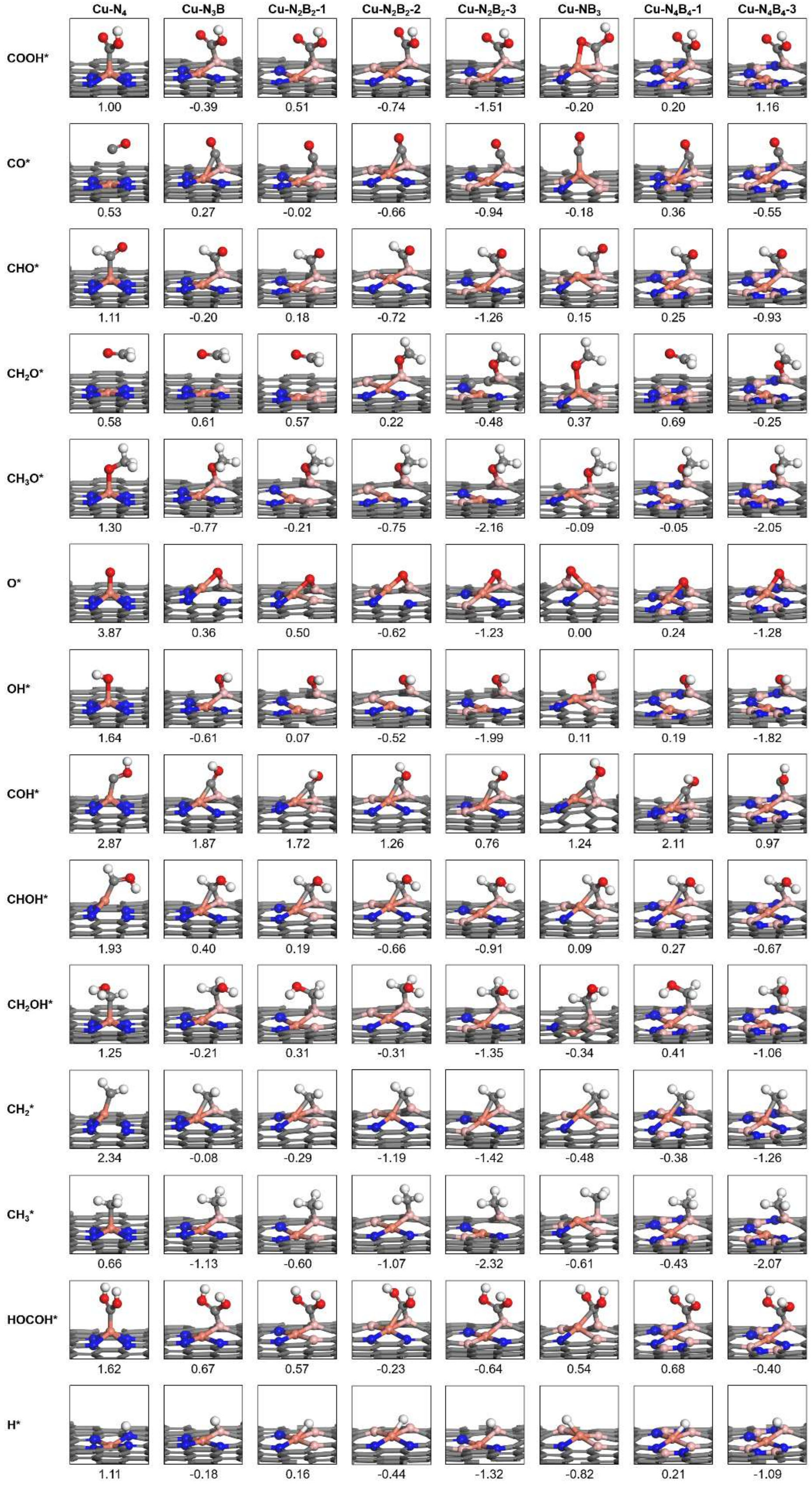

Supplementary Fig. 2 | The optimized adsorption configurations and energies (eV) of possible intermediates over considered Cu-N_xB_y sites, Cu (orange), N (blue), B (pink), C (gray), O (red), and H (white).

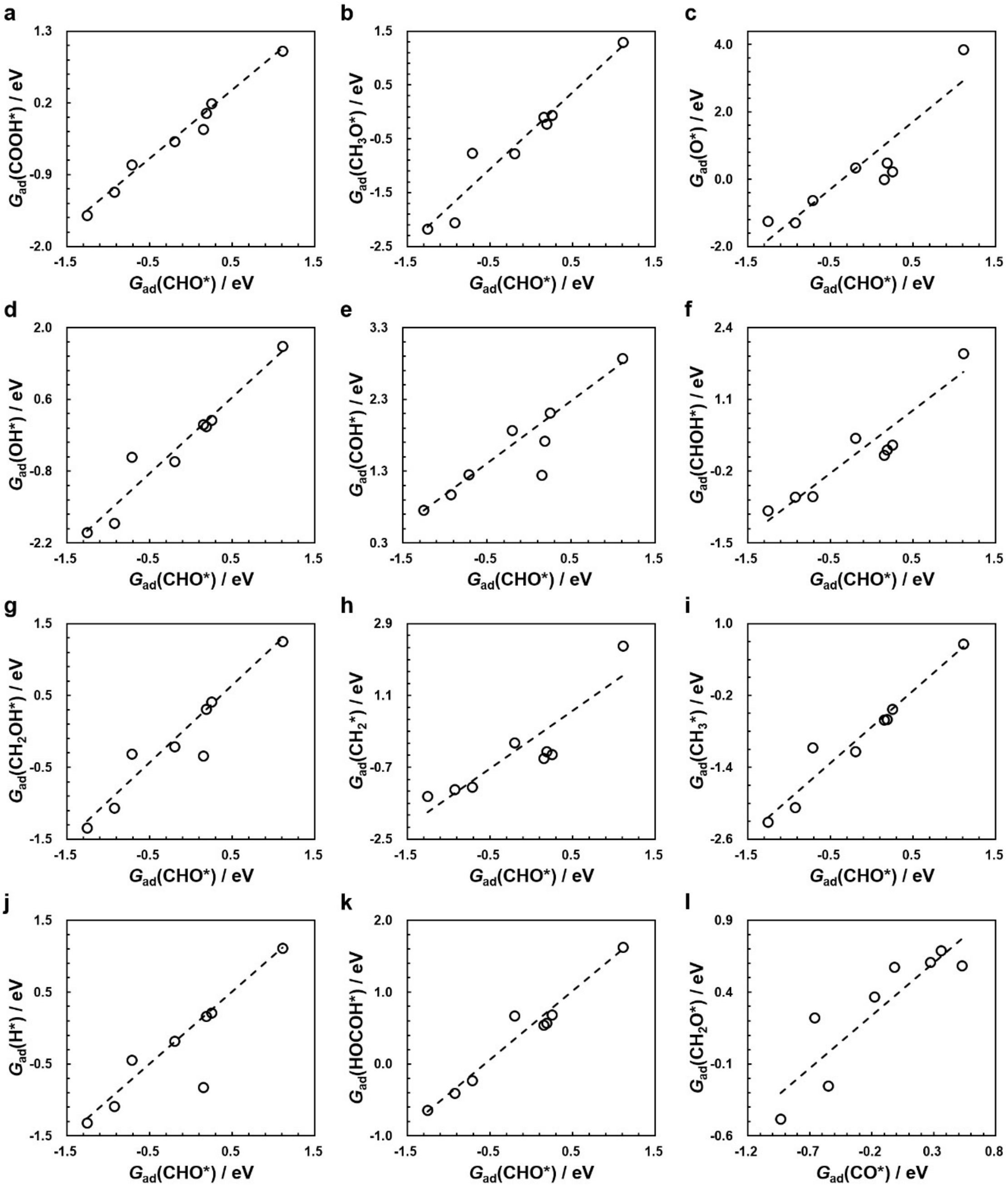

Supplementary Fig. 3 | The scaling relations over considered Cu-N_xB_y sites.

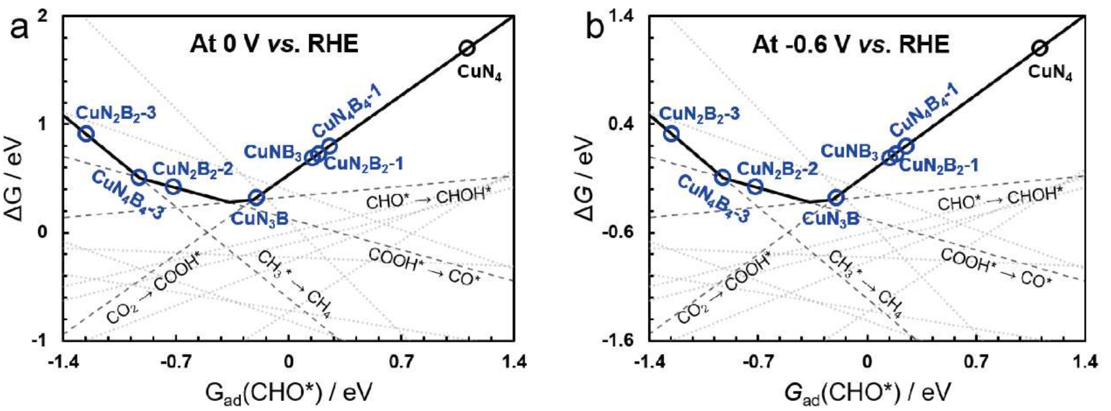

Supplementary Fig. 4 | Thermodynamic trend of Cu-N_xB_y with different B concentrations for CO_2RR to CH_4 at 0 V *vs.* RHE (**a**) and -0.6 V *vs.* RHE (**b**), as a function with G_{ad}(CHO*).

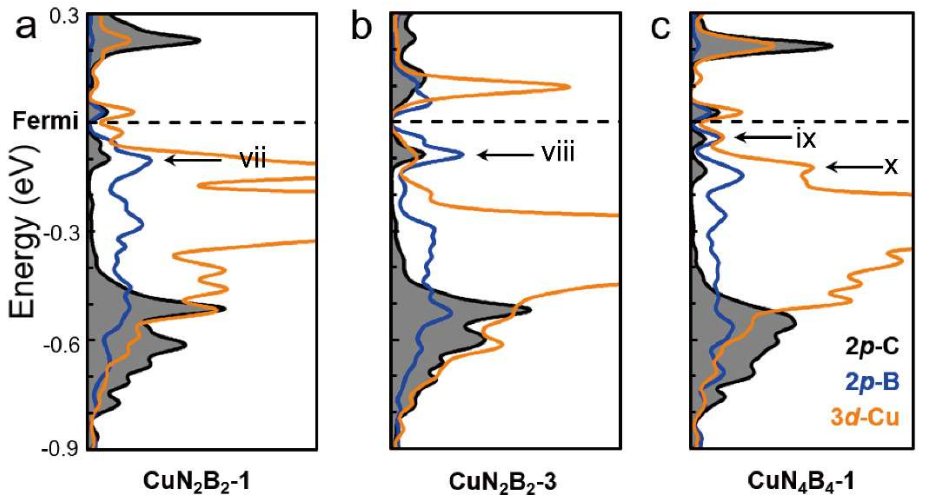

Supplementary Fig. 5 | a-c, Projected density of states (PDOS) for adsorbed CHO* over Cu-N_2B_2-1 **(a)**, Cu-N_2B_2-3 **(b)**, and Cu-N_4B_4-1 **(c)**, where the electronic states of 3*d*-Cu, 2*p*-C (of CHO*), and 2*p*-B are shown in orange, gray, and blue, respectively.

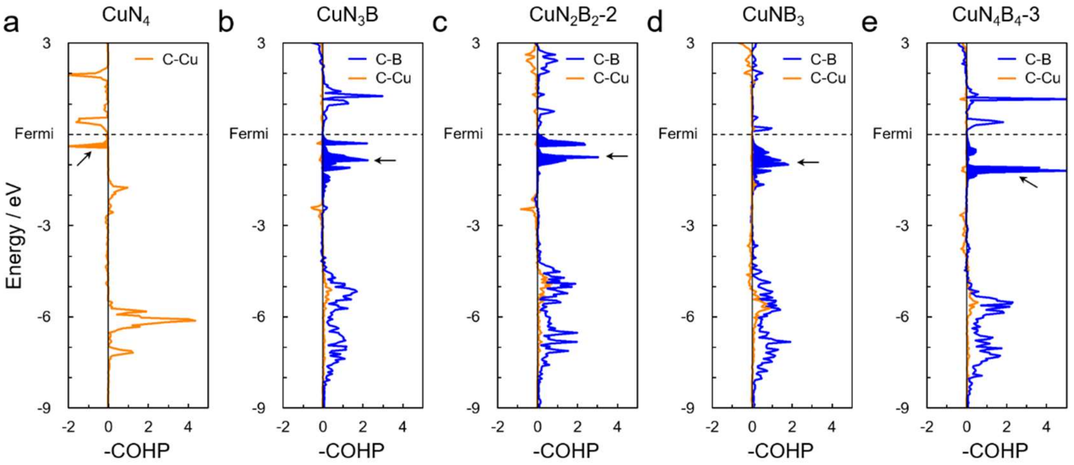

Supplementary Fig. 6 | a-e The analysis of crystal orbital Hamilton population (COHP) for adsorbed CHO* on CuN_4 (**a**), CuN_3B (**b**), CuN_2B_2-2 (**c**), $CuNB_3$ (**d**) and CuN_4B_4-3 (**e**), where the bonds of C-Cu and C-B are shown in orange and blue, respectively. The COHP analysis showed that, the intermediate CHO* adsorbs on CuN_4 through C atom bonding with Cu (C-Cu), where the antibonding states of C-Cu bond is partially occupied. With B substituting in Cu-N_4 motif, the C-B bonds are much stronger than C-Cu, indicating the enhancement of CHO* adsorption at Cu-N_xB_y sites. Besides, the marked peaks below Fermi level (**b-e**) refer to the strong electronic resonance between 2*p*-C (of CHO*) and 2*p*-B of CuN_xB_y sites.

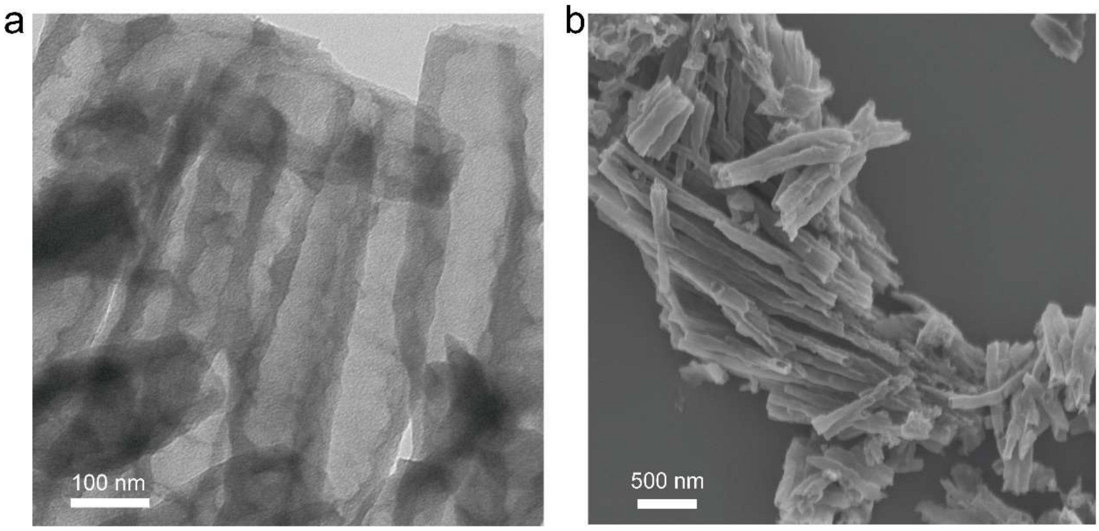

Supplementary Fig. 7 | TEM image (a) and SEM image (b) of NC-Cu. Both TEM and SEM images confirmed the tubular structure of NC-Cu.

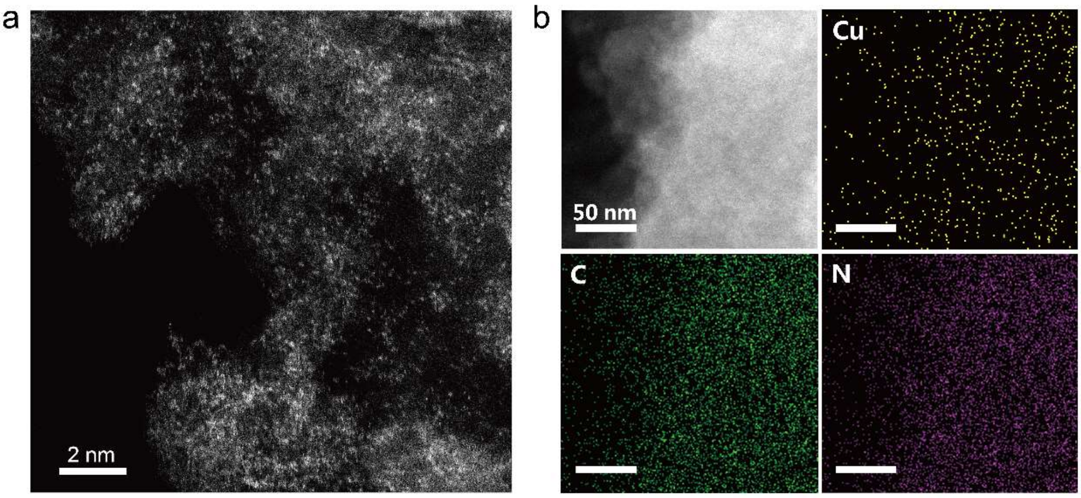

Supplementary Fig. 8 | HAADF-STEM image (a) and EDS elemental mapping (b) of NC-Cu. HAADF-STEM image verified the atomically dispersed copper atoms and EDX elemental mapping indicated the homogeneous distribution of Cu, C, and N in NC-Cu.

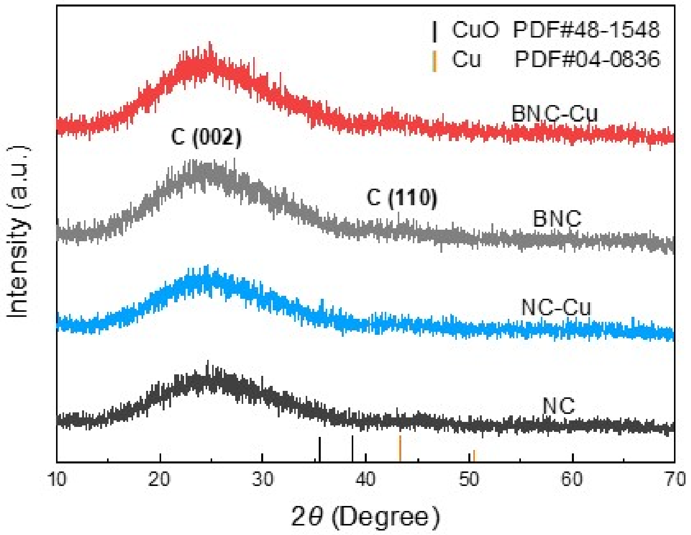

Supplementary Fig. 9 | XRD pattern of BNC-Cu and pure BNC. Both BNC-Cu and NC-Cu showed similar XRD pattern as pure-substrates. No visible peak ascribed to CuO or Cu could be seen.

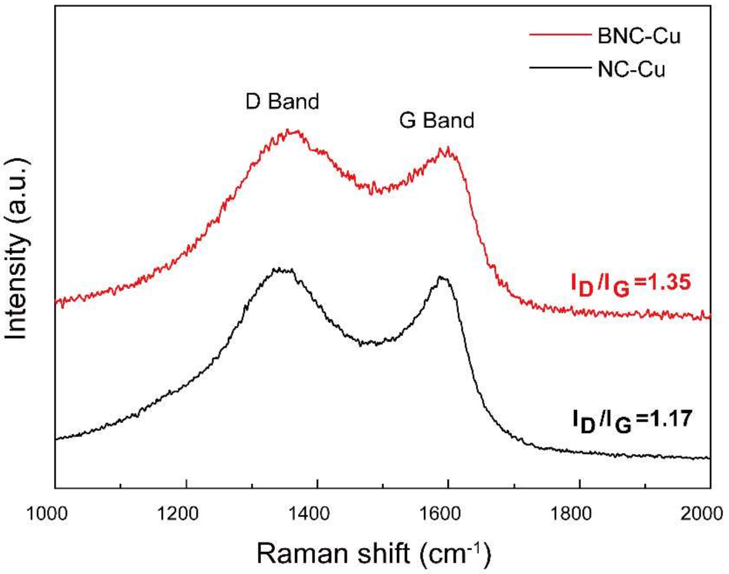

Supplementary Fig. 10 | Raman spectra of BNC-Cu and NC-Cu. Though both Raman spectra of BNC-Cu and NC-Cu showed typical feature of carbon materials, the I_D/I_G of such two materials still displayed obvious difference, signifying the introduction of B atoms into N-doped C matrix.

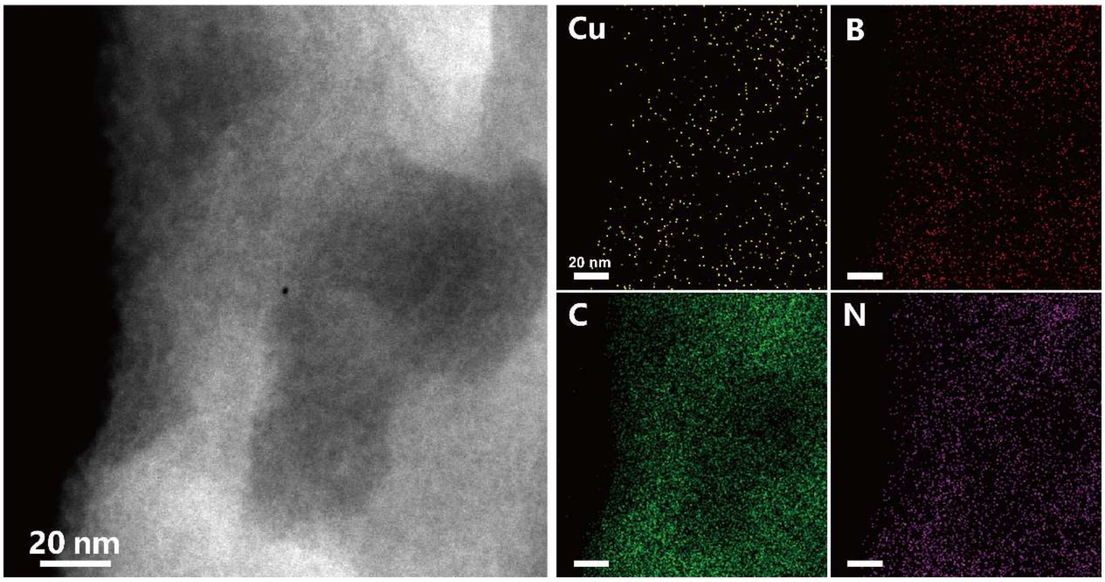

Supplementary Fig. 11 | Low-magnified STEM image of BNC-Cu and EDS elemental mapping towards the same region. Low-magnified STEM image also excluded the existence of copper nanoparticles. EDS elemental mapping in towards the same region indicated the homogeneous distribution of Cu, B, C, and N element.

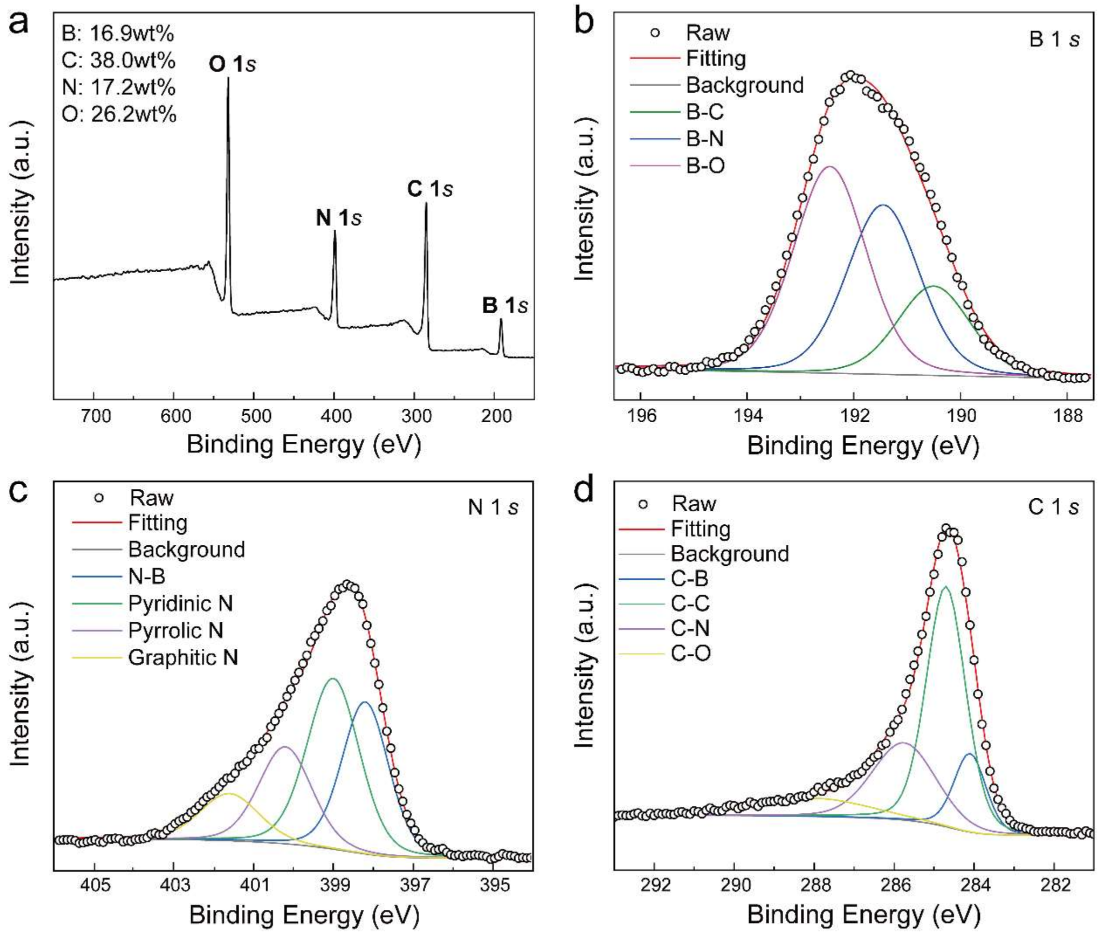

Supplementary Fig. 12 | High resolution XPS for BNC-Cu. a, XPS survey scan. **b-d,** B 1*s* **(b)**, N 1*s* **(c)**, and C 1*s* **(d)** spectra for BNC-Cu. According to the elemental composition of BNC-Cu as shown in XPS survey, large and compatible amounts of N and B atoms were successfully doped together into C matrix. C 1*s* spectra of XPS also showed signals of C-B and C-N bonds, consistent with the C *K*-edge XAS spectra.

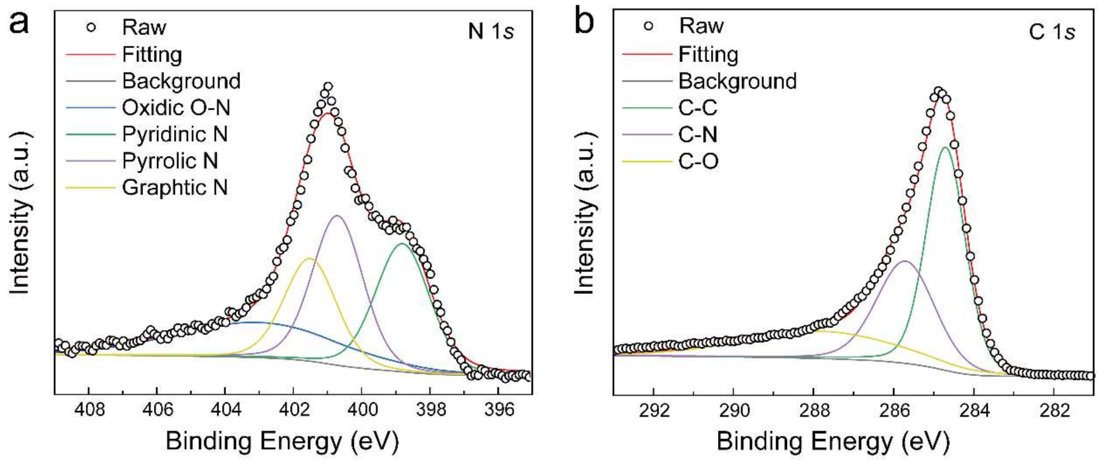

Supplementary Fig. 13 | High resolution XPS of N 1*s* (a) and C 1*s* (b) for NC-Cu. N 1*s* spectrum together with C 1*s* spectrum of NC-Cu proved that the support structure in NC-Cu was nitrogen doped carbon structure. More specifically, N 1*s* spectrum showed that more pyrrolic N species existed in NC-Cu catalysts, while there were more pyridinic N species in BNC-Cu.

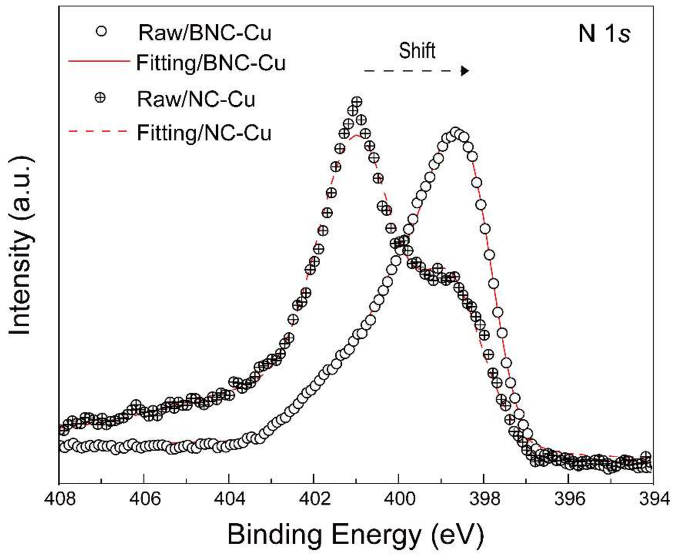

Supplementary Fig. 14 | Comparison of XPS N 1*s* spectra for BNC-Cu and NC-Cu. Red shift of N 1*s* spectrum between BNC-Cu and NC-Cu was attributed to different N species generated from general interaction between B and N atoms.

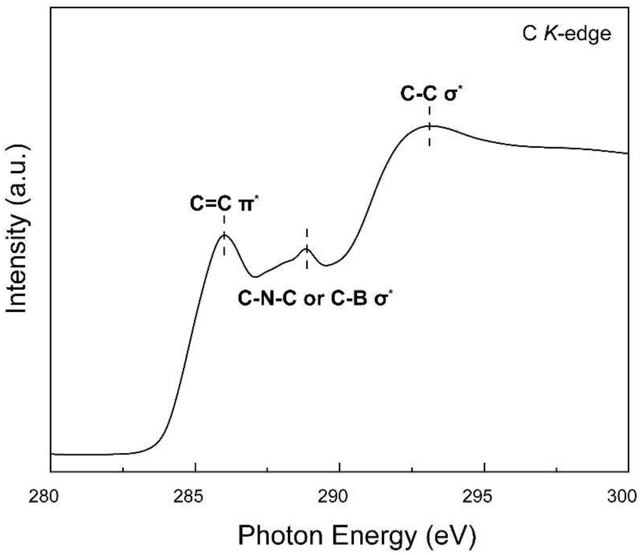

Supplementary Fig. 15 | C *K*-edge XANES spectra of BNC-Cu. C *K*-edge spectra of BNC-Cu showed characteristic peak of C-N, C-B, C-C, and C=C.

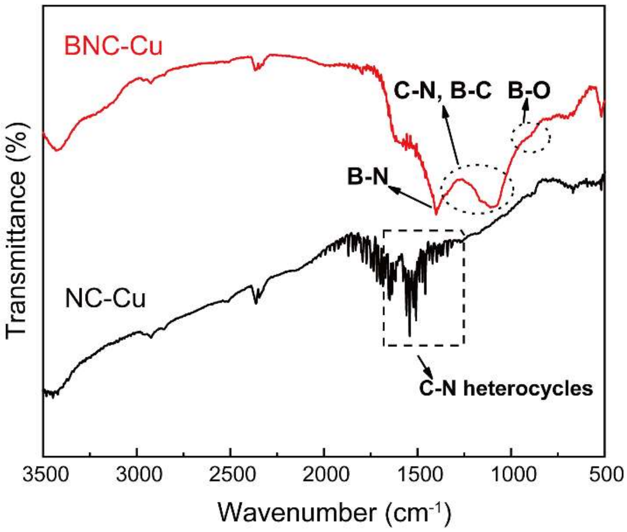

Supplementary Fig. 16 | FTIR spectra of BNC-Cu and NC-Cu. FTIR spectrum of BNC-Cu showed the characteristic peaks of B-N bonds around 1400 cm^{-1}. Peaks corresponding to C-N and C-B could also be seen from 1097 to 1238 cm^{-1}. A small peak at 890 cm^{-1} was ascribed to B-O bonds[1]. While FTIR spectrum of NC-Cu only showed typical peaks of C-N heterocycles[2].

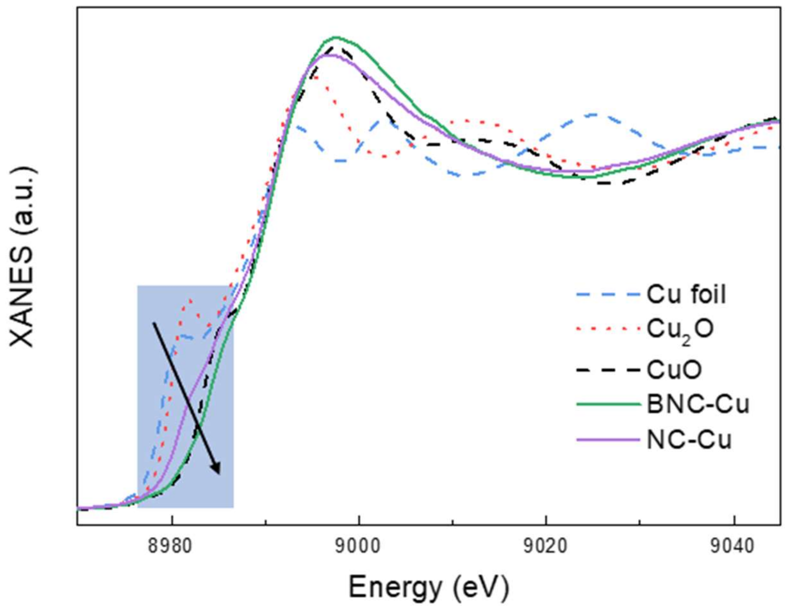

Supplementary Fig. 17 | Normalized XANES spectra at Cu *K*-edge of BNC-Cu, CuO, Cu_2O, and Cu foil. The white line of both BNC-Cu and NC-Cu was much higher than that of Cu foil, suggesting strong interaction between Cu atoms and substrates. The energy absorption threshold of NC-Cu was situated between that of Cu foil and CuO, indicating that Cu atoms in Cu-N_4 carried a partially positive charge between 0 and +2. While the energy absorption threshold of BNC-Cu was much closer to that of CuO, showing that the electronic properties of Cu single atoms were successfully tailored due to the different coordination environment of Cu atoms in BNC-Cu.

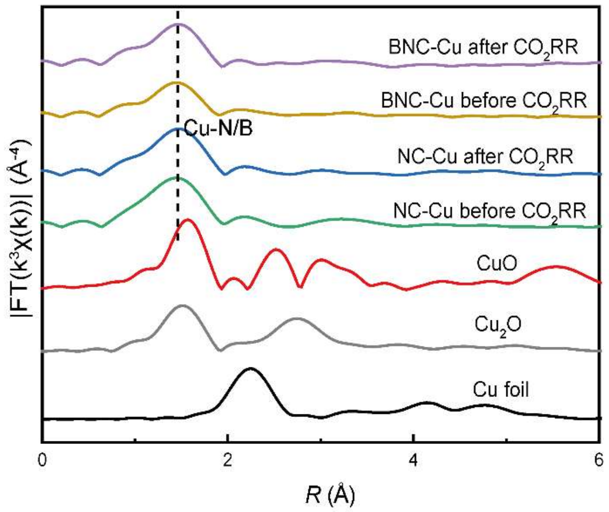

Supplementary Fig. 18 | Comparison of FT-EXAFS spectra at Cu *K*-edge for various samples. According to the FT-EXAFS spectra of Cu *K*-edge, no visible signal of Cu-Cu coordination could be seen in both BNC-Cu and NC-Cu catalysts, indicating the atomically dispersed Cu atoms in such two catalysts. FT-EXAFS failed to distinguish the Cu-B scattering peak from the Cu-N scattering peak, with both BNC-Cu and NC-Cu showed one intensity peak at similar radial distance of 1.46 Å, different from typical Cu-O scattering peak.

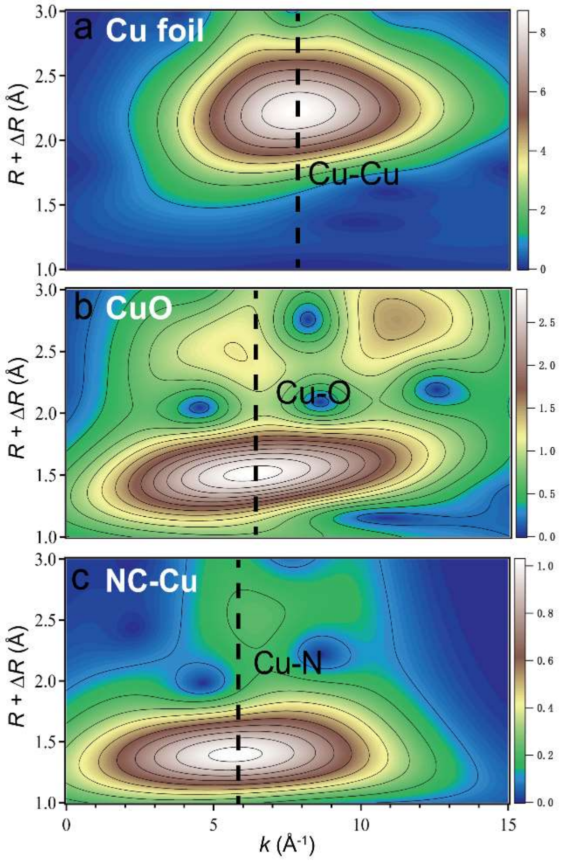

Supplementary Fig. 19 | Comparison of WT-EXAFS spectra for Cu foil (a), CuO (b), and NC-Cu (c). WT Spectra for Cu foil and CuO showed two intensity peaks at 7.90 $Å^{-1}$ and 6.40 $Å^{-1}$, attributed to typical Cu-Cu and Cu-O coordination, respectively. While WT spectrum for NC-Cu showed one intensity peak with smaller *k* than Cu-Cu and Cu-O coordination, corresponding to Cu-N coordination.

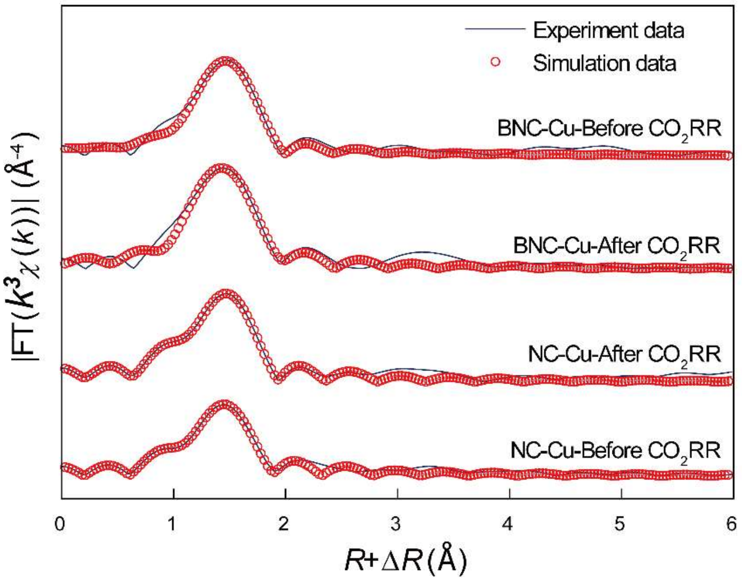

Supplementary Fig. 20 | FT-EXAFS spectra of BNC-Cu and NC-Cu before and after CO_2RR. Negligible difference could be seen before and after CO_2RR, demonstrating good electrochemical stability of BNC-Cu and NC-Cu.

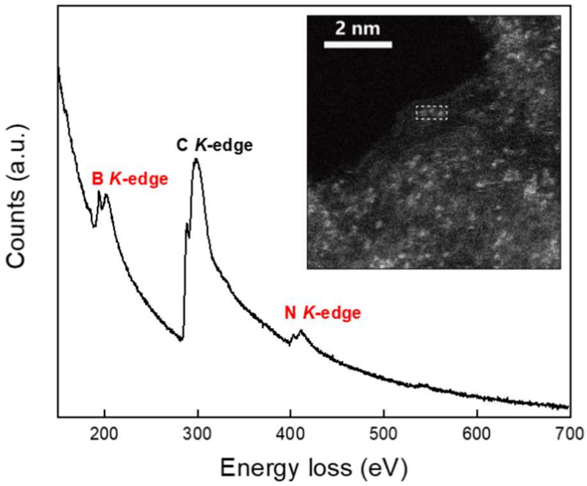

Supplementary Fig. 21 | EELS point spectrum for BNC-Cu. EELS point spectrum obtained at the edge of substrate (white box in HADDF-STEM image) on a small area, containing three Cu single atoms (bright dots), showed the colocation of B and N atoms.

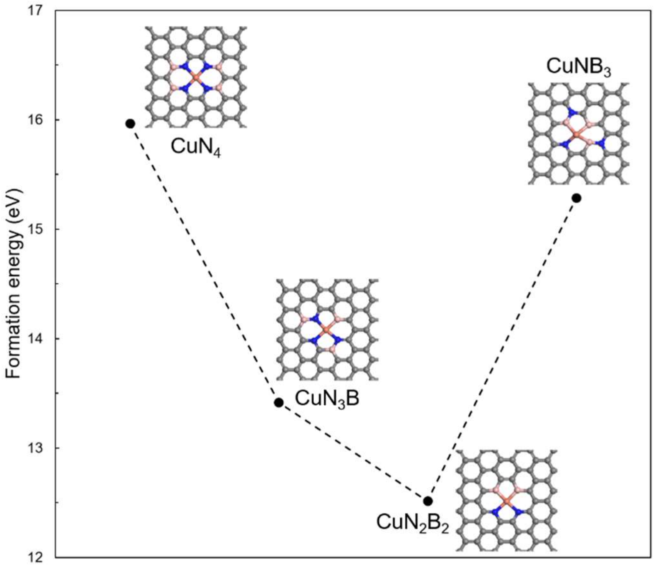

Supplementary Fig. 22 | Formation energy calculations among typical coordination structures. Given that the atomic ratio of N and B in BNC-Cu is very close and much higher than Cu from the XPS measurement, Cu-$N_{4-x}B_x$ (x=0–3) fragments are simulated with equal amount of N and B atoms (inset of **Supplementary Fig. 22**). The energies of Cu and C are referenced to metallic Cu and graphene, respectively. The chemical potential of N and B are calculated via the energy of HNO_3, H_3BO_3, NH_2CN and H_2O. Note that the absolute formation energies are tightly related to the reference whereas we focus on the relative stability of $CuN_{4-x}B_x$, so that the relative trend of positive formation energy is sufficient. Calculations of the formation energy among different coordination structures confirm that Cu-N_2B_2 is the most stable structure in BNC-Cu, with the lowest formation energy compared with other structures. Though the formation of other Cu-N_xB_y centers could not be completely excluded under a rapid and harsh pyrolysis condition, determined by the thermodynamic distribution, calculations of the formation energy among different coordination structures still confirmed Cu-N_2B_2 as the most stable structure in BNC-Cu, again validating the majority of Cu-N_2B_2 center, consistent with the XAS analysis.

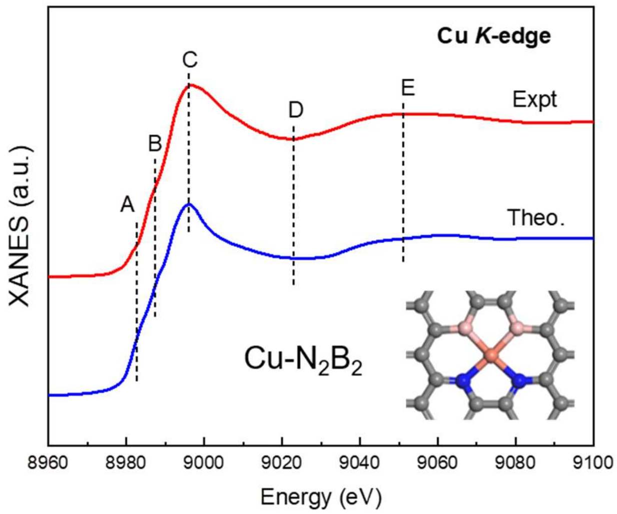

Supplementary Fig. 23 | Comparison between the theoretical XANES spectrum of Cu-N_2B_2 structure and experimental data of BNC-Cu. The calculation result for Cu-N_2B_2 model could best reproduce the main features of the experimental curve of BNC-Cu, verifying the EXAFS fitting result, while the subtle mismatch of experimental spectrum and simulation result was probably ascribed to other Cu-N_xB_y centers with a low proportion.

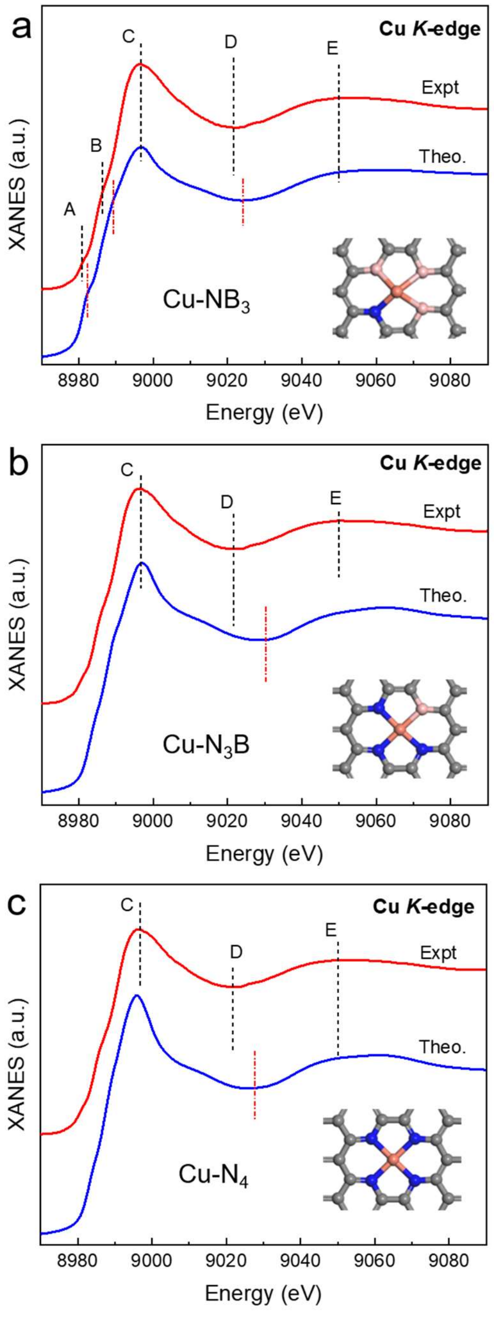

Supplementary Fig. 24 | Comparison between experimental data of BNC-Cu and the theoretical XANES spectrum of (a) $Cu\text{-}NB_3$, **(b)** $Cu\text{-}N_3B$, and **(c)** $Cu\text{-}N_4$.

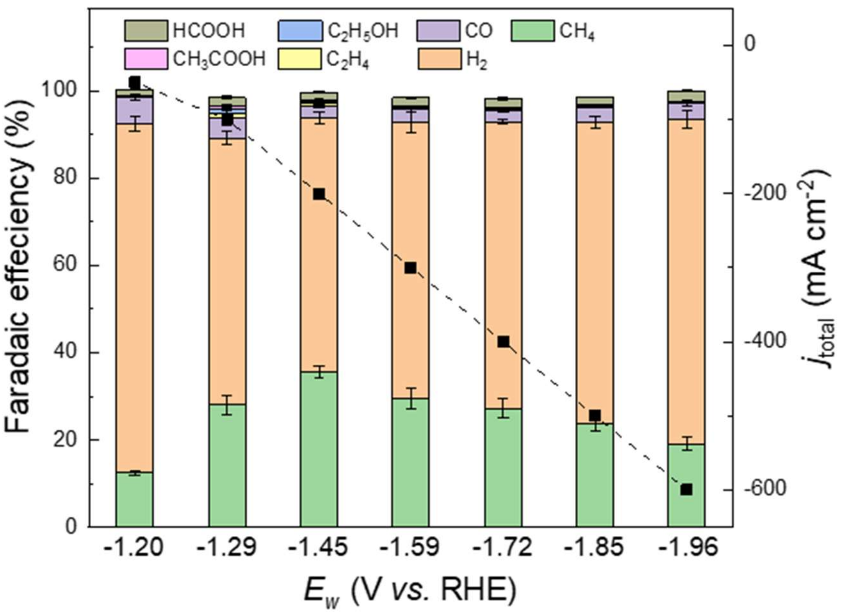

Supplementary Fig. 25 | Faradaic efficiency for various products of NC-Cu at various potentials. NC-Cu showed a high activity towards HER with FE of H_2 kept above 60% at varied current densities, and the FE of CH_4 rarely surpassed 30%. The error bars of FEs are calculated based on three independent measurements.

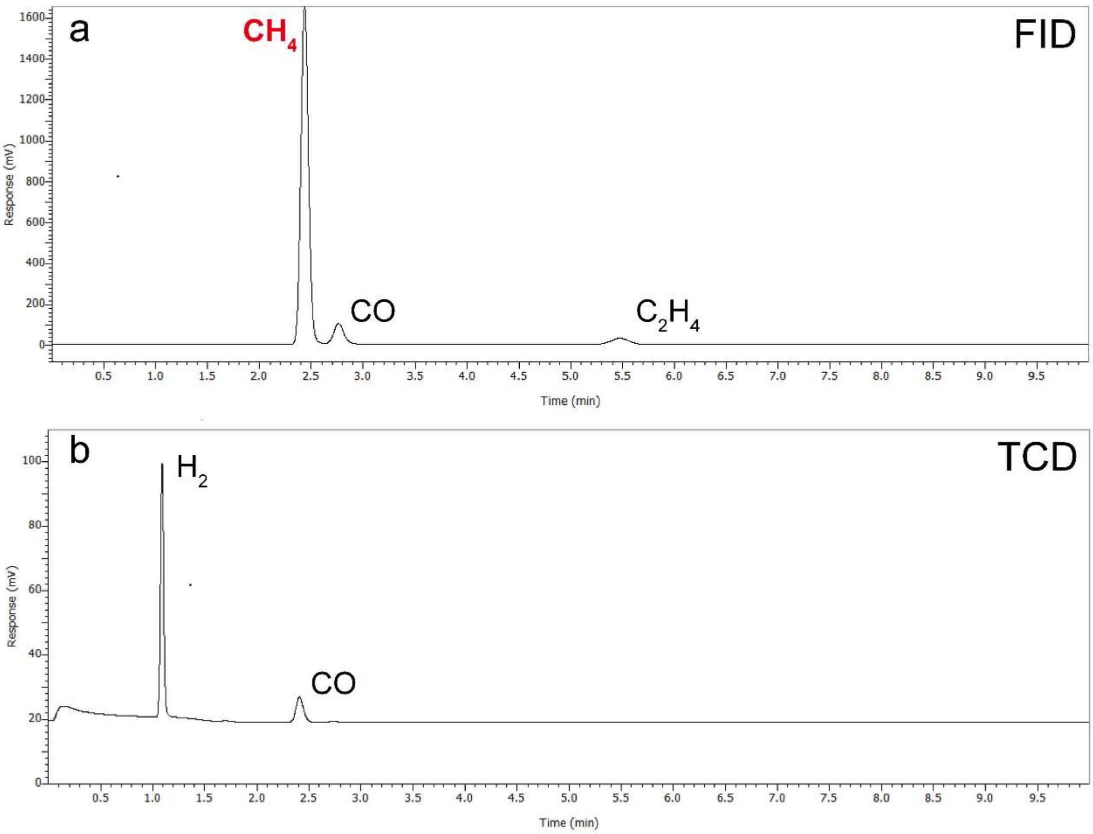

Supplementary Fig. 26 | Gaseous products at -400 mA cm^{-2} quantified *via* online GC analysis.

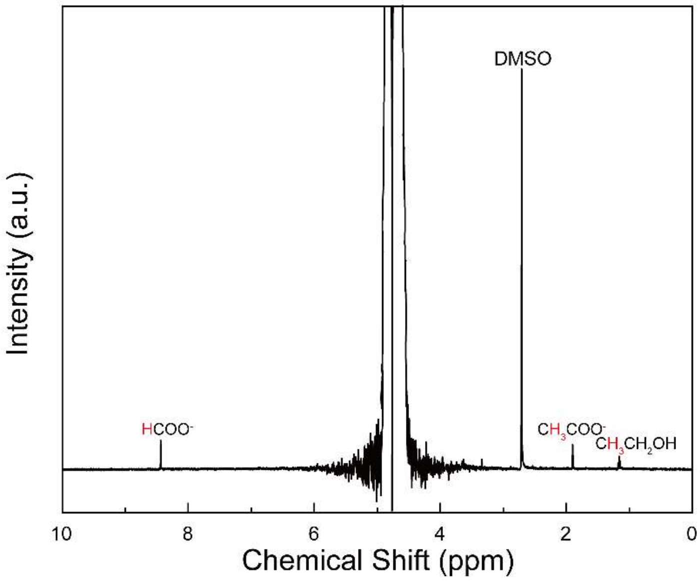

Supplementary Fig. 27 | ^{1}H Nuclear Magnetic Resonance (NMR) results for aqueous products at -400 mA cm^{-2}.

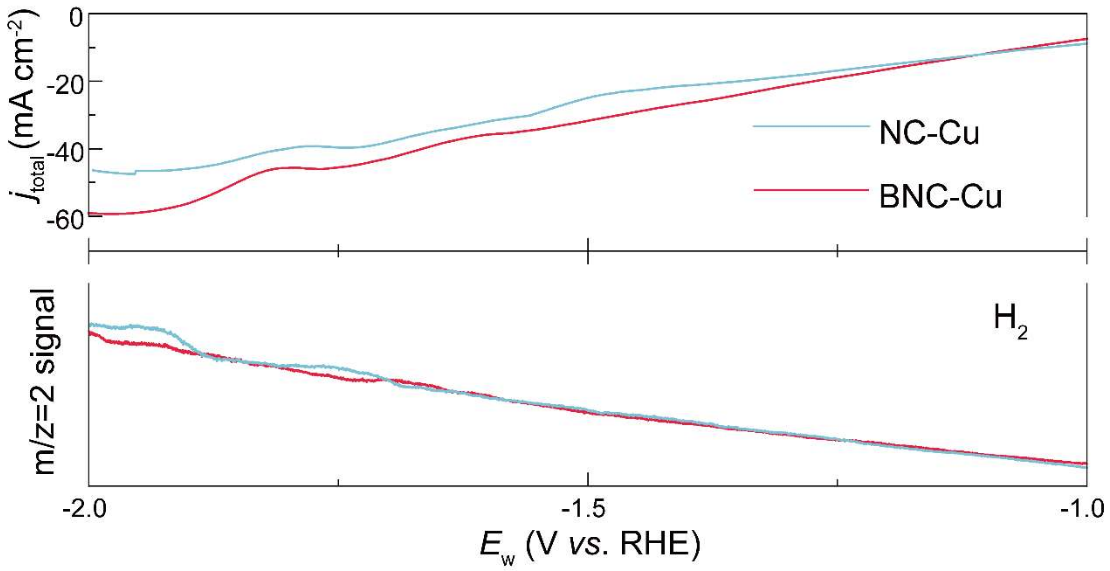

Supplementary Fig. 28 | Total current density and current ions signal of H_2 (m/z=2) for BNC-Cu and NC-Cu in DEMS test. BNC-Cu showed higher current density than NC-Cu from -1V *vs.* RHE to more negative potential, while m/z=2 signal which corresponds to H_2 produced showed negligible difference between BNC-Cu and NC-Cu, suggesting a preferential CO_2RR trend of BNC-Cu than NC-Cu.

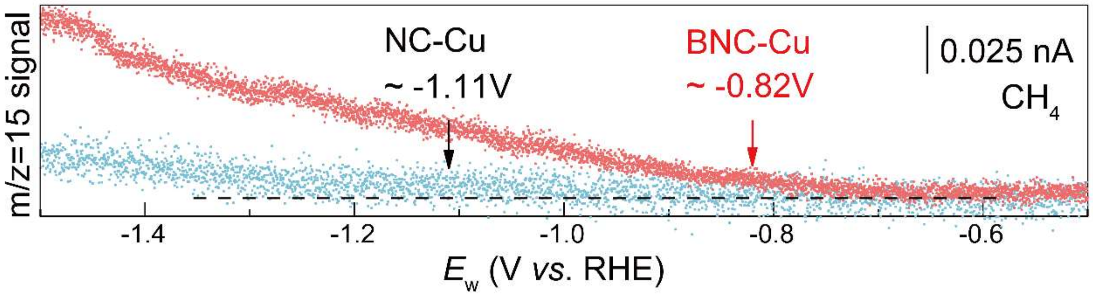

Supplementary Fig. 29 | Current ions signal of CH_4 for BNC-Cu and NC-Cu changing with applied potential. The above figure is a zoomed in view of the trends of CH_4 in **Fig. 3b**. The auxiliary dotted line in the figure represents the scribed line with current ion signal of 0. Defining the potential, where S/N=5, as the onset potential, then we extrapolated the CH_4 onset of -0.82 V *vs.* RHE for BNC-Cu and -1.11 V *vs.* RHE for NC-Cu.

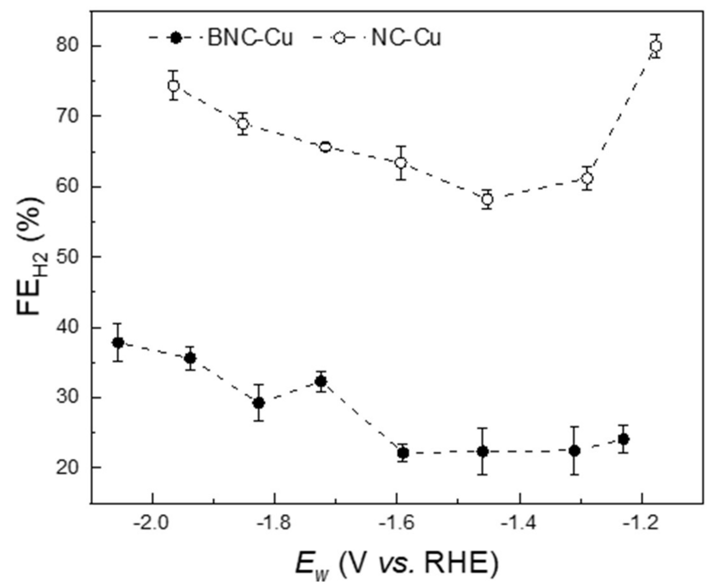

Supplementary Fig. 30 | Comparison of FE_{H2} for BNC-Cu and NC-Cu at various similar cathodic potentials. Compared with BNC-Cu, NC-Cu displayed a dominant activity towards HER with FE_{H2} > 60% at a wide potential range, showing a similar trend as previous works[3-5] due to weak adsorption of intermediates. The error bars of FE_{H2} are calculated based on three independent measurements.

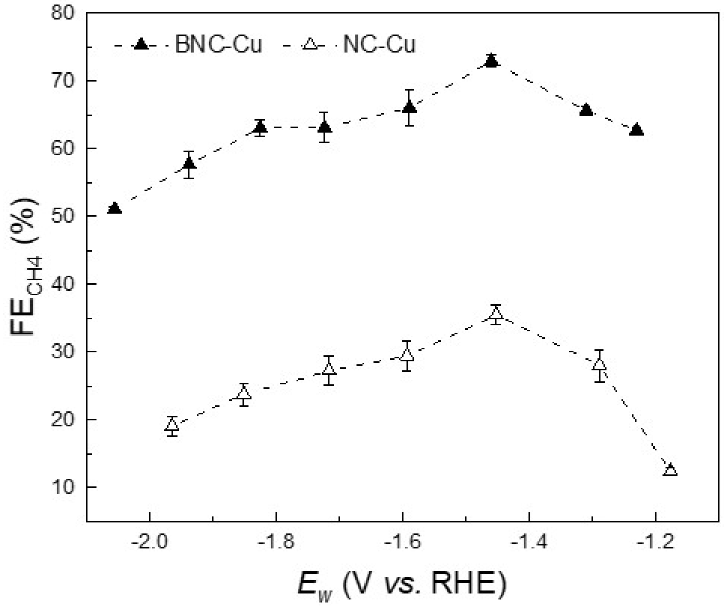

Supplementary Fig. 31 | Comparison of FE_{CH4} for BNC-Cu and NC-Cu at various similar cathodic potentials. FE_{CH4} of NC-Cu rarely surpassed 30% at all tested cathodic potentials, while FE_{CH4} of BNC-Cu basically kept above 60% at similar potential range. The error bars of FE_{CH4} are calculated based on three independent measurements.

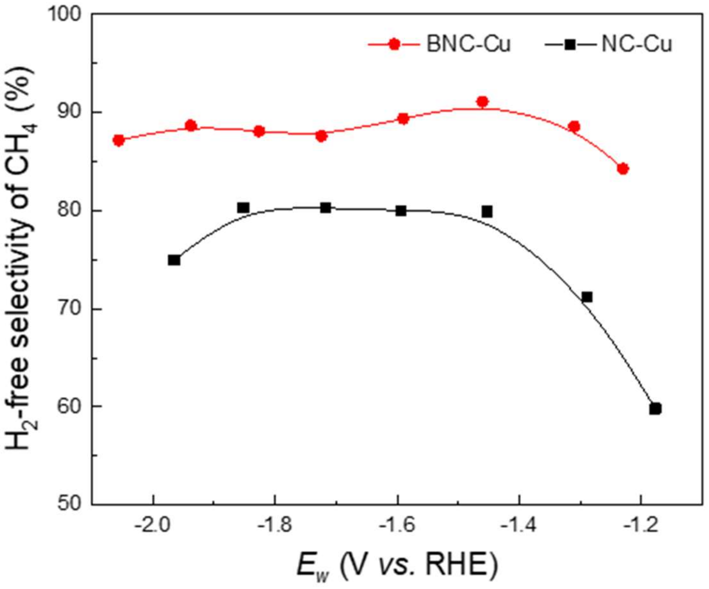

Supplementary Fig. 32 | H_2-free selectivity of CH_4 in CO_2RR products at different current densities for BNC-Cu and NC-Cu. H_2 free selectivity of CH_4 is defined as the proportion of electrons consumed by methane to the total electrons consumed by all carbonaceous product. The selectivity of CH_4 among CO_2RR products for BNC-Cu kept above NC-Cu for a wide potential range and a larger difference could be seen at higher potentials, demonstrating better intrinsic activity for CH_4 production of BNC-Cu.

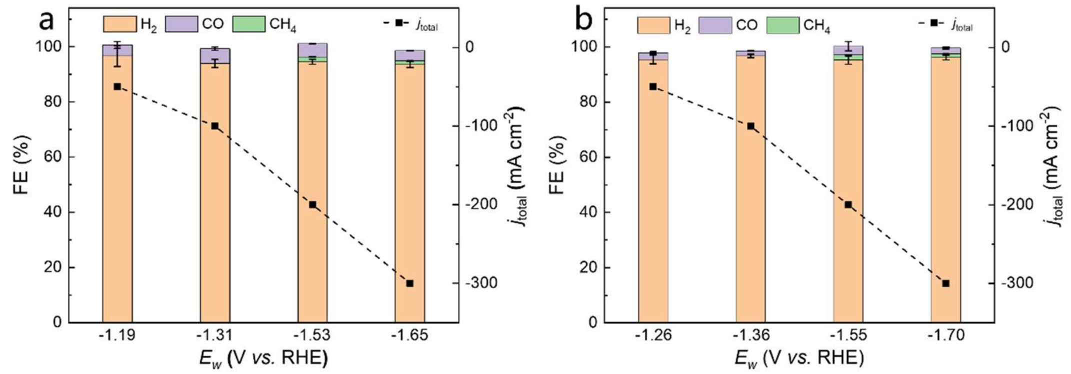

Supplementary Fig. 33 | Faradaic efficiencies for various products of (a) BNC and (b) NC at different applied potentials. HER dominated in both pure substrates (BNC and NC) at various potentials while CH_4 only showed up at pretty high potential with FE less than 2%. The error bars of FEs are calculated based on three independent measurements.

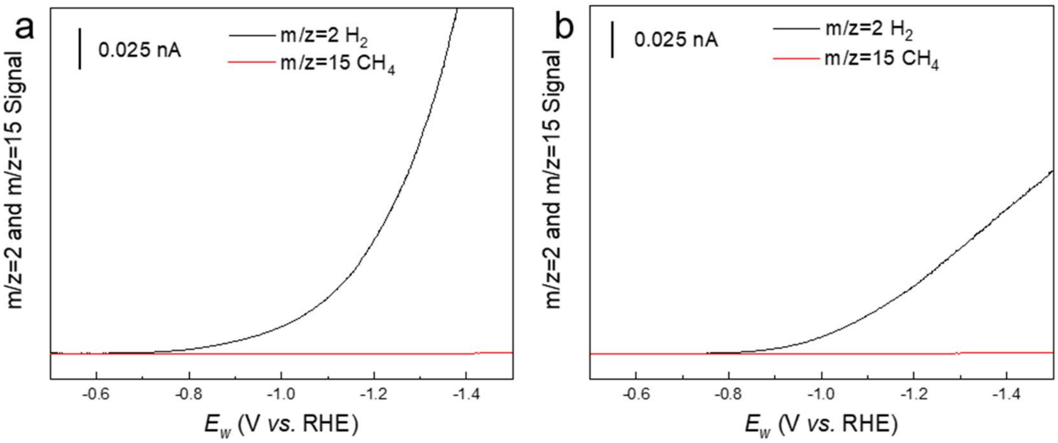

Supplementary Fig.34 | *In-situ* DEMS data of (a) BNC and (b) NC. Negligible CH_4 production could be seen for both BNC and NC with potential range of -0.5 V to -1.5 V *vs.* RHE.

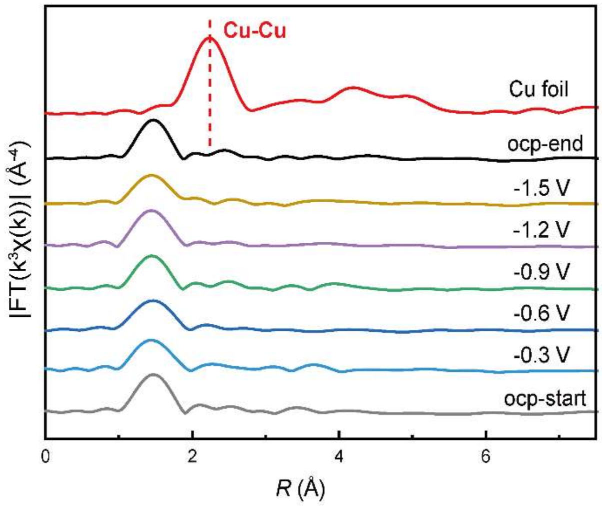

Supplementary Fig. 35 | FT-EXAFS spectra of NC-Cu at Cu *K*-edge at different applied potentials. No visible signal of Cu-Cu coordination could be seen when applied with different negative potentials ranging from -0.3 to -1.5 V *vs.* RHE, verifying that the dominant Cu sites in NC-Cu remained as Cu single atoms during CO_2 electrolysis.

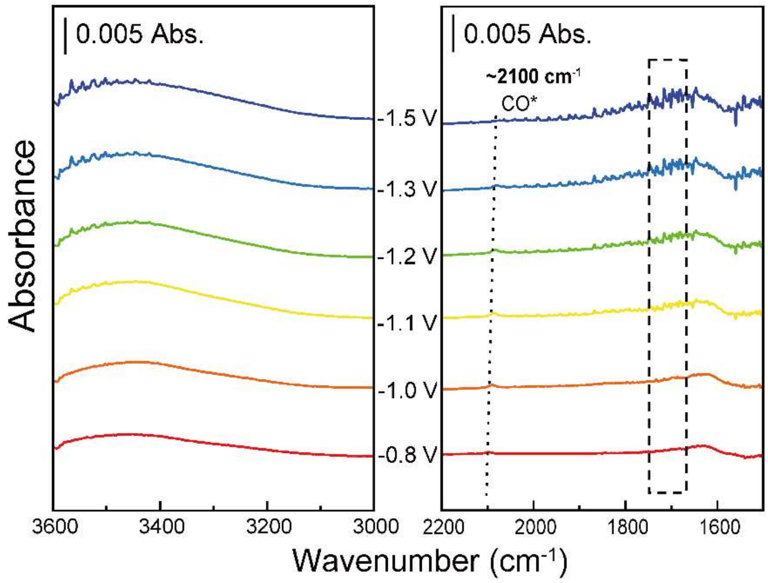

Supplementary Fig. 36 | *In-situ* ATR-IR spectra for NC-Cu. Such spectra did not show any visible CHO* peak, but a CO* peak around 2100 cm^{-1} which kept steady with elevated applied potential, indicating that the CO* to CHO* was sluggish on NC-Cu. Two peaks ascribed to water (around 1650 cm^{-1} and 3400 cm^{-1}) also did not show much tendency to increase with potential, suggesting that NC-Cu was less affinitive with water molecules.

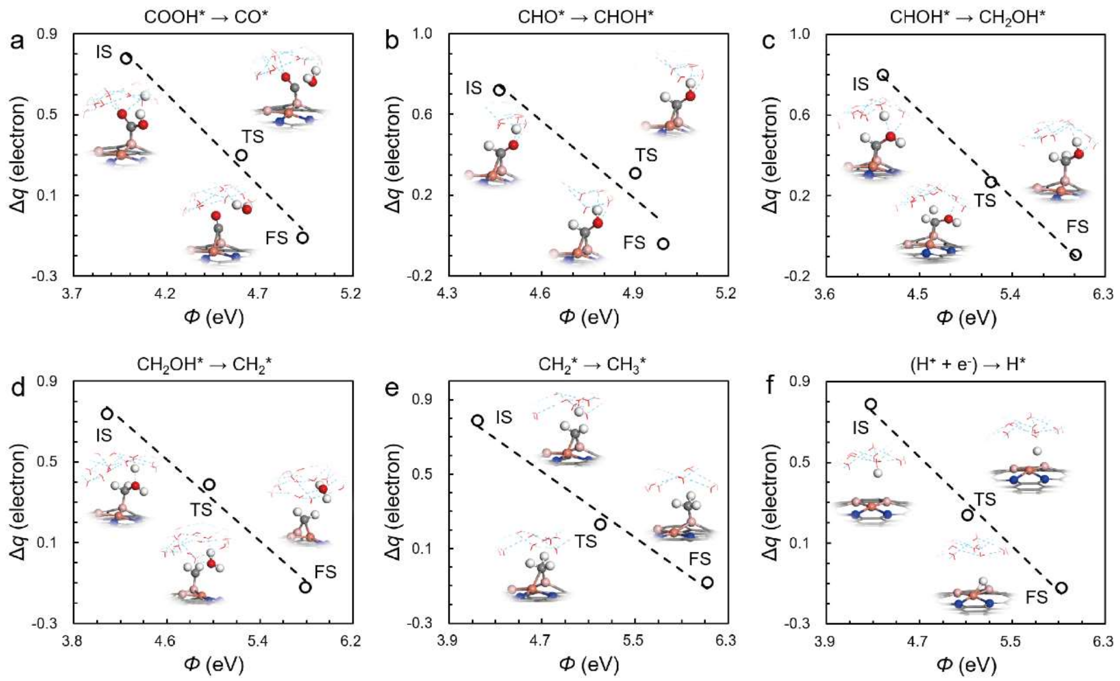

Supplementary Fig. 37 | Calculated charge transfer (Δq) and Φ on electrochemical interface at the initial states (IS), transition states (TS), and final states (FS) for different steps over Cu-N_2B_2-2.

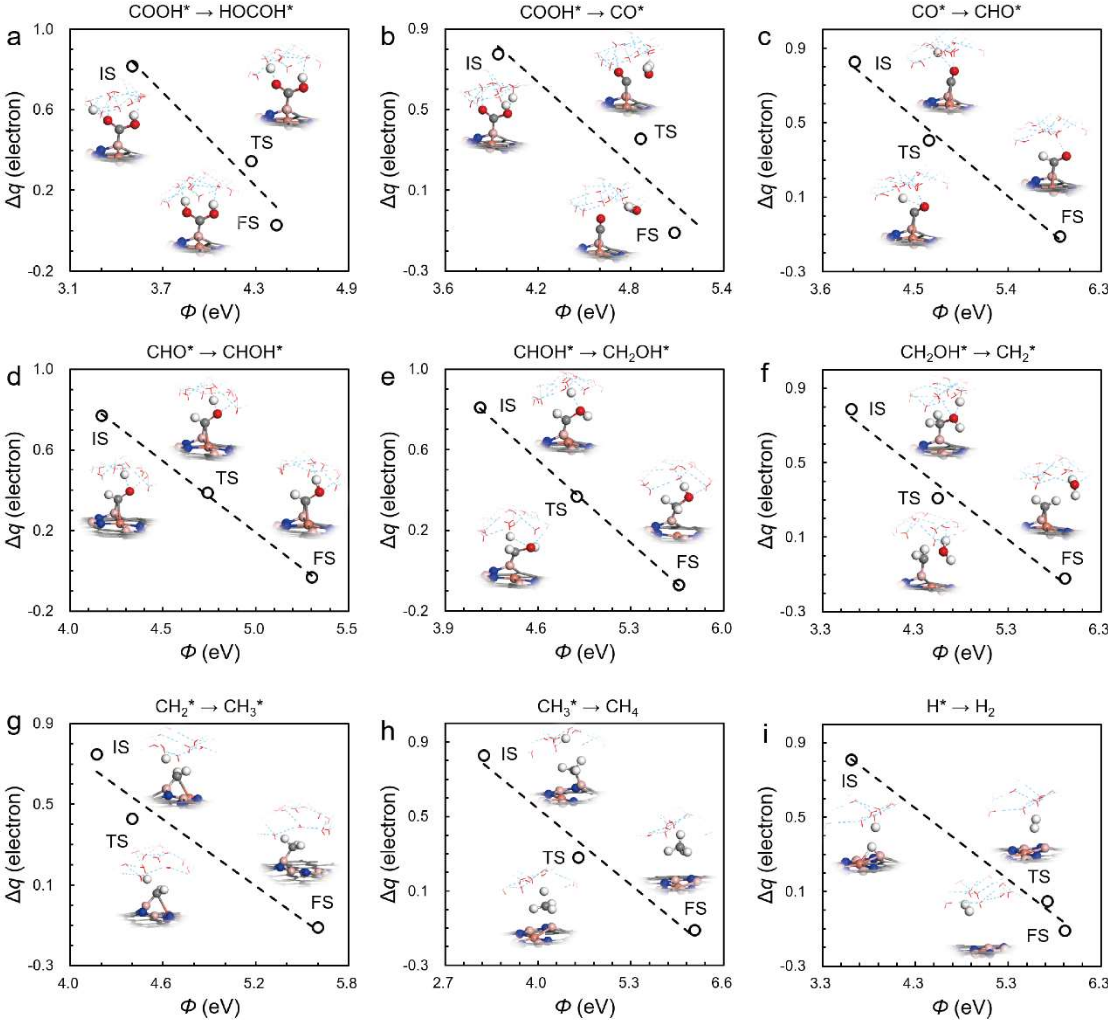

Supplementary Fig. 38 | Calculated charge transfer (Δq) and Φ on electrochemical interface at the initial states (IS), transition states (TS), and final states (FS) for different steps over Cu-N_4B_4-3.

Supplementary Table 1 | EXAFS fitting parameters of the best-fit models at the Cu *K*-edge for various samples.

Sample	Shell	N^{a}	$R(\text{Å})^{b}$	$\sigma^2(\text{Å}^2)^{c}$	$\Delta E_0(\text{eV})^{d}$	*R* factor
Cu foil	Cu-Cu	12	2.54	0.0088	4.4	0.0006
BNC-Cu Before CO_2RR	Cu-N	2.1	1.91	0.0036	-2.8	0.0007
	Cu-B	2.2	2.11	0.0034		
BNC-Cu After CO_2RR	Cu-N	2.0	1.89	0.0033	-3.8	0.0008
	Cu-B	2.2	2.10	0.0047		
NC-Cu Before CO_2RR	Cu-N	3.5	1.93	0.0127	-2.5	0.0001
NC-Cu After CO_2RR	Cu-N	3.8	1.94	0.0076	-1.1	0.0001

^{a}N: coordination numbers; ^{b}R: bond distance; $^{c}\sigma^2$: Debye-Waller factors; $^{d}\Delta E_0$: the inner potential correction. *R* factor: goodness of fit. S_0^2 was set to 0.814 for Cu, according to the experimental EXAFS fit of Cu foil reference by fixing coordination number as the known crystallographic value.

Supplementary Table 2 | EXAFS fitting parameters of other excluded models at the Cu *K*-edge for BNC-Cu.

Sample	Shell	N^a	R(Å)^b	σ^2(Å^2)^c	ΔE_0(eV)^d	R factor
BNC-Cu Before CO_2RR	Cu-N	4	1.96	0.0064	-1.1	0.0039
BNC-Cu-Before CO_2RR	Cu-N<br>Cu-B	3<br>1	1.94<br>2.12	0.0049<br>0.0042	-1.3	0.0013
BNC-Cu-Before CO_2RR	Cu-N<br>Cu-B	1<br>3	1.88<br>2.04	0.0053<br>0.0008	-5.6	0.0263
BNC-Cu-Before CO_2RR	Cu-B	4	2.01	0.0019	-7.1	0.0647
BNC-Cu-After CO_2RR	Cu-N	4	1.95	0.0064	-2.1	0.0038
BNC-Cu-After CO_2RR	Cu-N<br>Cu-B	3<br>1	1.92<br>2.09	0.0040<br>0.0044	-2.7	0.0025
BNC-Cu-After CO_2RR	Cu-N<br>Cu-B	1<br>3	1.88<br>2.01	0.0033<br>0.0026	-6.6	0.0325
BNC-Cu-After CO_2RR	Cu-B	4	1.98	0.0014	-9.7	0.0695

$^a N$: coordination numbers; $^b R$: bond distance; $^c \sigma^2$: Debye-Waller factors; $^d \Delta E_0$: the inner potential correction. R factor: goodness of fit. S_0^2 was set to 0.814 for Cu, according to the experimental EXAFS fit of Cu foil reference by fixing coordination number as the known crystallographic value.

Reference

1. Zhang, X. et al. Methanol conversion on borocarbonitride catalysts: Identification and quantification of active sites. *Sci. Adv.* **6**, eaba5778 (2020).
2. Lv, C. et al. Defect Engineering Metal-Free Polymeric Carbon Nitride Electrocatalyst for Effective Nitrogen Fixation under Ambient Conditions. *Angew. Chem. Int. Ed.* **57**, 10246-10250 (2018).
3. Zheng, W. et al. Atomically Defined Undercoordinated Active Sites for Highly Efficient CO_2 Electroreduction. *Adv. Funct. Mater.* **30**, 1907658 (2020).
4. L, Jiao. Et al. Single-Atom Electrocatalysts from Multivariate Metal–Organic Frameworks for Highly Selective Reduction of CO_2 at Low Pressures. *Angew. Chem. Int. Ed.* ***59***, 20589 (2020).
5. Chang, Q. et al. Metal-Coordinated Phthalocyanines as Platform Molecules for Understanding Isolated Metal Sites in the Electrochemical Reduction of CO_2. *J. Am. Chem. Soc.* **144**, 16131-16138 (2022).
